# Single-cell atlas of the tumor immune microenvironment across syngeneic murine models

**DOI:** 10.3389/fimmu.2025.1676581

**Published:** 2025-11-14

**Authors:** Jia Wang, Bin Jiang, Minjuan Deng, Han Yan, Pei Zhang, Wei Jin, Zhirong Shen

**Affiliations:** Translational Discovery, Research and Medicine, BeOne Medicines, Beijing, China

**Keywords:** tumor immune microenvironment, syngeneic murine models, single-cell atlas, anti-PD-1therapy, interferon-stimulated gene-high (ISGhigh) monocyte subset, neutrophil depletion

## Abstract

The tumor immune microenvironment plays a critical role in tumor progression and responses to immunotherapy. Nevertheless, its cellular complexity and heterogeneity remain incompletely understood. In this study, we employed high-resolution single-cell RNA sequencing on CD45+ immune cells isolated from ten syngeneic murine tumor models, representing seven distinct cancer types under treatment-naïve conditions, thereby enabling a comprehensive profiling of tumor-infiltrating immune cells. We identified seven principal immune cell populations and provided an in-depth characterization of T cells, NK/innate lymphoid cells, dendritic cells, monocytes/macrophages, and neutrophils. Cross-species analyses further delineated conserved immune cell states and transcriptomic features within the T cell and monocyte/macrophage compartments that are shared across syngeneic models and human tumors. To investigate the functional relevance of the predominant monocyte/macrophage compartment and the notable presence of neutrophils in syngeneic tumors, we evaluated responses to anti-PD-1 therapy across various models and analyzed the enrichment of monocyte/macrophage subsets in tumors that responded to treatment. Furthermore, we conducted neutrophil depletion experiments using anti-Ly6G antibodies, administered both as monotherapy and in combination with PD-1 blockade. Remarkably, an interferon-stimulated gene-high (ISG^high^) monocyte subset was significantly enriched in models responsive to anti-PD-1 therapy. Neutrophil depletion resulted in variable antitumor effects across models but failed to enhance the efficacy of PD-1 blockade. In summary, our single-cell profiling offered a detailed atlas of the immune microenvironment across multiple syngeneic mouse tumor models, thereby enabling rational model selection for immuno-oncology studies. We uncovered an ISG^high^ monocyte subset enriched in anti-PD-1 responsive models, and showed the context-dependent effects of neutrophil depletion on tumor immunity and immunotherapy, underscoring the heterogeneity and functional divergence of immune cell sublineages.

## Introduction

The tumor microenvironment (TME) is a complex and heterogeneous ecosystem, comprising the extracellular matrix, non-immune cells (such as endothelial and stromal cells), and a diverse array of immune cells that collectively constitute the tumor immune microenvironment (TIME). Mounting evidence highlights the critical role of the TIME in tumor progression, recurrence, metastasis, and, notably, in modulating responses to immunotherapies (1).

Accordingly, immunotherapies that target the inhibitory receptors expressed by immune cells have yielded remarkable therapeutic benefits in clinical practice. However, even the most successful immune checkpoint blockade (ICB) strategies encounter significant challenges related to treatment resistance. For example, anti-PD-1/PD-L1 therapies only demonstrate an average durable objective response rate of merely 25% in solid malignancies. In melanoma, the most responsive cancer type, approximately 1/4 to 1/3 responders experience relapse after a period of treatment (2). Retrospective studies have identified the characteristics of the TIME as key determinants of therapeutic response and resistance, underscoring the imperative for a more profound understanding of its cellular composition and molecular features (1–3).

Murine syngeneic tumor models, established by implanting tumor cell lines into genetically identical mouse strains, are widely used in preclinical cancer immunology due to their immunocompetence and intact immune systems. Previous studies have characterized the TIME in various syngeneic models, revealing differences in gene expression, immune composition, and the functional roles of specific immune cell populations (see Discussion) (4–12). However, a comprehensive cross-model analysis of the TIME - particularly of less abundant or less well-characterized immune subsets - remains limited.

Here, we utilized high-resolution single-cell RNA sequencing (scRNA-seq) to systematically characterize the baseline immune landscape across ten commonly used murine syngeneic tumor models, providing detailed insights into immune cell infiltration and heterogeneity. We delineated features conserved across human and mouse TIMEs and resolved the discrete contributions of macrophage and neutrophil subpopulations to antitumor immunity and responsiveness to anti-PD-1 therapy, thereby underscoring the translational value of this atlas.

## Materials and methods

### Animal models

Three immunocompetent mouse strains - Balb/C, C57BL/6N and FVB - were utilized in this study. All animals were female, aged 6 to 8 weeks, and sourced from Beijing Vital River Laboratory Animal Technology Co., Ltd. Mice were housed in a vivarium under specific pathogen-free conditions, with up to five animals per cage. All procedures involving animals were carried out with the approval of the Institutional Animal Care and Use Committee (IACUC) of BeOne Medicines.

### Cell lines

Ten distinct murine tumor cell lines were used to establish syngeneic models. The origins and culture conditions of these cell lines were detailed in **Table 1**. The conditions for cell implantation in mice were summarized in **Table 2**.

**Table 1:** Information about the murine tumor cell lines used in this study.

Cell line	Source	Culture medium
4T1	ATCC (CRL-2539)	RPMI-1640 medium (Gibco, Cat# 22400-105) supplemented with 10% (v/v) fetal bovine serum (Gibco, Cat# 10099-141) and 100 ug/ml of penicillin and streptomycin (Gibco, Cat# 15140-122)
CT26.WT	ATCC (CRL-2638)	
GL261	NCI (E-172-2015)	
Pan02	NCI (E-050-2016)	
LL2	ATCC (CRL-1642)	DMEM (Gibco, Cat# 11965092) supplemented with 10% (v/v) fetal bovine serum (Gibco, Cat# 10099-141) and 100 ug/ml of penicillin and streptomycin (Gibco, Cat# 15140-122)
B16F10	ATCC (CRL-6475)	
EMT6	ATCC (CRL-2755)	Waymouth medium (Gibco, Cat# 11220035) supplemented with 15% (v/v) fetal bovine serum (Gibco, Cat# 10099-141) and 100 ug/ml of penicillin and streptomycin (Gibco, Cat# 15140-122)
MMTV-PyMT	JAX mice (#002374)	DMEM/F12 medium (Gibco, Cat# 11320-082) supplemented with 1X Insulin-Transferrin-Selenium (ITS) (Gibco, Cat# 51500-056), 10% (v/v) fetal bovine serum (Gibco, Cat# 10099-141), and 100 ug/ml of penicillin and streptomycin (Gibco, Cat# 15140-122)
MC38	Kerafast (ENH204)	DMEM (Gibco, Cat# 11965092) supplemented with 10 mM HEPES (Gibco, Cat# 15630-080), 50 µg/mL gentamicin (PhytoTechnology, G3350-10ML), 0.1 mM NEAA (Gibco, Cat# 11140-050), 10% (v/v) fetal bovine serum (Gibco, Cat# 10099-141), and 100 ug/ml of penicillin and streptomycin (Gibco, Cat# 15140-122)
Renca	ATCC (CRL-2947)	RPMI-1640 medium (Gibco, Cat# 22400-105) supplemented with 1 mM sodium pyruvate (Corning, Cat# R25-000-Cl), 2 mM L-glutamine (Gibco, Cat# 25030-081), 0.1 mM NEAA (Gibco, Cat# 11140-050), 10% (v/v) fetal bovine serum (Gibco, Cat# 10099-141), and 100 ug/ml of penicillin and streptomycin (Gibco, Cat# 15140-122)

**Table 2:** Implantation parameters for syngeneic tumor models.

Cell line	Mouse strain	Cell number	Implanted position
4T1	Balb/C	3X10^5	mammary fat pad
EMT6	Balb/C	5X10^4	mammary fat pad
MMTV-PyMT	FVB	1X10^6	mammary fat pad
CT26.WT	Balb/C	1X10^5	rear right flank
MC38	C57BL/6N	1X10^6	rear right flank
GL261	C57BL/6N	1X10^7	rear right flank
Renca	Balb/C	1X10^6	rear right flank
LL2	C57BL/6N	3X10^5	rear right flank
B16F10	C57BL/6N	3X10^5	rear right flank
Pan02	C57BL/6N	1X10^7	rear right flank

### Single-cell sorting and library preparation

Tumors were harvested when they reached a volume of approximately 250–300 mm^3^. For each model, three tumors were collected, mechanically dissociated, and resuspended in 2.35 mL of RPMI 1640 medium supplemented with 100 µL of Enzyme D, 50 µL of Enzyme R, and 12.5 µL of Enzyme A (Miltenyi Biotec, Cat# 130-096-730). Tissue dissociation was performed using the gentleMACS™ Octo Dissociator with Heaters (Miltenyi Biotec, Cat# 130-096-427) according to the manufacturer’s program (37C_m_TDK_1).

Following dissociation, cell suspensions were filtered through a 70 μm mesh and washed with fluorescence-activated cell sorting (FACS) buffer (1% FBS in PBS). Cells were centrifuged at 500 × g for 5 minutes and resuspended in 500 μL of FACS buffer. For flow cytometry analysis, cells were stained with PerCP-Cy5.5 anti-mouse CD45 (BD Biosciences, clone 30-F11, Cat# 550994) and Fixable Viability Stain 450 (BD Biosciences, Cat# 562247). Following staining, viable CD45+ cells were isolated via FACS using a BD FACSAria™ SORP cell sorter (BD Biosciences, configured with 5 lasers (355nm, 405 nm, 488 nm, 561nm, and 640 nm) and 16 fluorescence detectors). Post-sorting reanalysis confirmed that >80% of cells used for downstream scRNA-seq were viable, as determined by the exclusion of Fixable Viability Stain 450-positive (non-viable) cells. CD45+ viable cells were washed in PBS and resuspended at a concentration of 1 × 10^6^ cells/mL. Single-cell suspensions were subsequently loaded onto a Chromium Controller (10x Genomics, Pleasanton, CA) using the Single Cell 3’ Library and Gel Bead Kit v3 (10x Genomics) for droplet-based encapsulation and library preparation.

### Efficacy evaluation in syngeneic mouse models

#### Anti-PD-1 response

To assess the efficacy of anti-PD-1 treatment in each model, mice bearing tumors were randomly assigned to treatment groups based on tumor volume or body weight. Mice received intraperitoneal (i.p.) injections of either an anti-mouse PD-1 antibody (clone Ch15mt, 3 mpk, produced by BIODURO on behalf of BeOne Medicines) or vehicle control (PBS solvent) once a week as monotherapy, initiated when tumor size reached 100–200 mm³. Body weight and tumor volume were measured biweekly. Tumor volume (mm³) was calculated using the formula: V = 0.5 (a x b²), where *a* and *b* represent the tumor’s long and short diameters, respectively. Mice were euthanized via carbon dioxide inhalation if tumor volume exceeded 2000 mm³, tumors became ulcerated, or body weight loss exceeded 20%. All outcome assessments and data analysis were performed by researchers blinded to group assignments.

#### *In vivo* neutrophil depletion

To evaluate the role of neutrophils in antitumor efficacy, mice were administered intraperitoneal (i.p.) injections of an anti-mouse Ly6G antibody (Bio X Cell, clone 1A8, Cat# BE0075-1) at a dose of 50 μg in 100 μL PBS or an isotype control once daily, starting on Day1 after grouping. Mice were euthanized using carbon dioxide once their tumor volume reaches ≥2000 mm^3^, the tumor became ulcerated, or body weight loss exceeds 20%. Neutrophil depletion efficiency was assessed by flow cytometry after 2 days of treatment with the anti-Ly6G antibody. For combination therapy studies, the anti-PD-1 antibody was administered as described above, starting on Day1 after grouping. Group sizes for each model were as follows: CT26.WT (n = 10), and EMT6 (n = 10) per group.

### Flow cytometry for neutrophil quantification

Neutrophil abundance was assessed by flow cytometry using the same protocol described for single-cell sorting. Cells were stained with eFluor™ 506 (eBioscience, Cat# 65-0866-18, diluted 1:1000), BV786-CD45 (Biolegend, clone 30-F11, Cat# 103149, diluted 1:800, final concentration 0.25 μg/mL), FITC-CD19 (BD Biosciences, clone 1D3, Cat# 553785, diluted 1:200, final concentration 2.5 μg/mL), FITC-CD3e (BD Biosciences, clone 145-2C11, Cat# 553062, diluted 1:200, final concentration 2.5 μg/mL), FITC-CD335 (BD Biosciences, clone 29A1.4, Cat# 560756, diluted 1:200, final concentration 2.5 μg/mL), APC-CD11b (BD Biosciences, clone M1/70, Cat# 561690, diluted 1:400, final concentration 0.5 μg/mL), PerCP-Cy5.5-Ly6G and Ly6C (BD Biosciences, clone RB6-8C5, Cat# 552093, diluted 1:400, final concentration 0.5 μg/mL) and PE/Cy7-CD115 (Biolegend, clone AFS98, Cat# 135524, diluted 1:200, final concentration 1 μg/mL) to identify neutrophil populations. The samples were incubated in the dark at 4 °C for 30 minutes, washed twice with PBS, and then resuspended in 200 μL of PBS. Each sample was analyzed on a Cytek Aurora full spectrum flow cytometer (Cytek Biosciences, configured with 3 lasers (405 nm, 488 nm, and 640 nm) and 38 fluorescence channels (16V-14B-8R)), with acquisition of no fewer than 10,000 live CD45+ cell events per sample. Data were analyzed using SpectroFlo software (version 3.0.3). Group sizes for each model were as follows: CT26.WT (n = 5) and EMT6 (n = 4).

### scRNA-seq data processing and cell type annotation

scRNA-seq was performed on CD45+ immune cells isolated from 30 tumor samples representing 10 syngeneic murine tumor models, with each model replicated in triplicate using individual animals. Raw sequencing data were processed using the Cell Ranger Single-Cell Software Suite v3.1.0 and aligned to the mm10 mouse reference genome.

The resulting gene expression matrices were imported into Seurat (version 5.1.0) (13) for downstream analysis. Quality control filtering excluded cells with <500 UMIs, <250 detected genes, or a gene-to-UMI ratio < 10^0.8. Cells with >10% mitochondrial gene content, potential doublets (identified via DoubletFinder (14) with optimized pK), or extreme values (>40,000 UMIs or >6,000 genes) were also removed. Genes expressed in fewer than 10 cells were excluded from further analysis.

Remaining cells were normalized using Seurat’s “Log Transform” function. The top 2,000 highly variable genes were selected for canonical correlation analysis (CCA)-based data integration (15) and principal component analysis. Clustering was performed using the “FindNeighbours” and “FindClusters” functions and visualized via uniform manifold approximation and projection (UMAP).

Major immune cell clusters emerged during unsupervised clustering at a resolution of 0.1. We assessed canonical immune marker gene expression across clusters and merged those with highly similar expression patterns. The final seven principal immune cell populations were annotated based on the following markers: *Cd3e* (T cells), *Ncr1* (NK cells), *Cd68* (monocytes/macrophages), *Csf3r* (neutrophils), *Siglech* (plasmacytoid dendritic cells), *Fscn1*, *Cd209a*, and *H2-DMb2* (conventional dendritic cells), *Ms4a2* (mast cells), and *Ms4a1* (B cells). For T cells, NK cells, dendritic cells, monocytes/macrophages, and neutrophils, we refined initial lineage assignments by iteratively increasing clustering resolution to reveal heterogeneous substructures. For each subcluster, we performed differential expression analysis with Seurat’s FindMarkers function (Wilcoxon rank-sum test) and single-cell gene set enrichment analysis with RunScGSEA function (category C5, GO: BP). Subclusters that exhibited at least three significantly differentially expressed genes relative to other subclusters (Benjamini-Hochberg-adjusted *p*-value < 0.05; average log2 fold-change > 0.5) were retained as separate clusters. As reference panels, we additionally applied automated annotation with SingleR against two mouse immune-relevant reference atlases (ImmGenData and MouseRNAseqData). Final assignments were further supported by clustering tree analysis and biologically relevant marker genes and pathways.

### Developmental trajectory analysis

To infer cellular state transitions and lineage relationships, we applied the Monocle2 (16) algorithm. The RNA expression matrix derived from the cluster-annotated Seurat object was normalized and converted into a CellDataSet object. Differentially expressed genes with a false discovery rate (*q*-value) < 0.01 from each cluster were used to order cells along pseudotime. Cellular trajectories were reconstructed using Monocle2’s default dimensionality reduction and cell ordering parameters, enabling the inference of potential differentiation pathways and transitional states within immune cell populations.

### Signature scoring

Gene signature scores were computed using the “AddModuleScore” function in Seurat. For each cell, the average expression of genes within a defined signature was calculated, and the aggregated expression of matched control gene sets was subtracted to yield the final module score. Signatures originally derived from human datasets were mapped to murine homologs based on gene symbol concordance. Genes lacking direct murine counterparts were excluded from analysis.

*T cell-related gene signatures* (17):

- Cytotoxicity: *Gzmb*, *Prf1*, *Fasl*
- Exhaustion: Pdcd1, Havcr2, Tigit, Lag3, Ctla4
- Stemness: *Tcf7*, *Sell*, *Il7r*, *Lef1*

*NK cell and ILC1-related gene signatures* (18–20):

- NK (Robinette2015): *Klra3, Klra10, Klra9, Irf8, Eomes, Klrg1, Scimp, Itgam, Cym, Serpinb9b, Klra1, Car5b, Cmklr1, Zeb2, Khdc1a*
- ILC1 (Robinette2015): *Trgv3, Trgv2, Il7r, Tmem176b, Il2ra, Cxcr6, Socs2, Ckb, Gpr114, Tmem176a, Podnl1, Gpr97, St6galnac3, Tmem154, Cdon, Atp8a2, Slc27a6*
- NK (Björklund2016): *Gzmb, Nkg7, Klrd1, Eomes, Itgax, Fcgr3a, Prf1, Gzma, Irf8, Slamf7, Ccl4, Fam49a, Gzmk, Aoah, Gzmc, Zmat4, Cd160, 1700025G04Rik, Ccr1, Styk1, Cdhr1*
- ILC1 (Björklund2016): *Sit1, Cd3d, Cd3g, Cd4, Cd6, Trav13-1, Cd5, Cd27, Cd8a, Trav4-1, Gzmk, Trbv5, Adtrp, Trav9-2*
- Intratumoral NK: *Itga1, Eef1g, Serbp1, Top2a, Pa2g4, Aldoa, Plac8, Gzmb, Irf8, Gm5559, Pgk1, Ldha, Mif, Pkm2, Tpi1, Gzmc, Xcl1, Lgals1*
- NK activation: *Bcl2, Ccl3, Ccl4, Ccr5, Cd69, Cxcl10, Foxk1, Gbp4, Gzmb, Icam1, Ifih1, Ifng, Il12rb1, Il12rb2, Il2ra, Irf1, Irf7, Irf8, Klf11, Klf13, Klrg1, Myd88, Nfil3, Nfkbib, Nfkbiz, Notch1, Nr4a1, Nr4a2, Nr4a3, Socs1, Socs3, Stat1, Stat2, Stat4, Tbx21*

*Mo/Mφ-related gene signatures* (21, 22):

- IFN-TAMs: *Ccl2, Ccl7, Ccl8, Cd274, Cxcl9, Cxcl10, Cxcl11, Ifit1, Ifit2, Ifit3, Ifitm1, Ifitm3, Il7r, Isg15, Nos2, Rsad2, Tnfsf10, Stat1*
- Inflam-TAM: *Cxcl1, Cxcl2, Cxcl3, Cxcl5, Cxcl8, Ccl20, Ccl3l1, Il1rn, Il1b, G0s2, Inhba, Spp1*
- Angio-TAMs: *Arg1, Adam8, Bnip3, Mif, Slc2a1*
- LA-TAMs: *Acp5, Apoc1, Apoe, C1qa, C1qb, C1qc, Ccl18, Ccl8, Cd163, Cd206, Cd36, Cd63, Ctsb, Ctsd, Ctsl, Cxcl9, Fabp5, Folr2, Gpnmb, Lgals3, Macro, Mrc1, Trem2*
- Reg-TAMs: *Apoe, Arg1, C1qa, Ccl2, Cd63, Clec4d, Cx3cr1, Gpnmb, Hilpda, Hmox1, Il7r, Mrc1, Pf4, Spp1, Trem2, Vegfa, Itga4*
- Prolif-TAM: *Cdk1, Mki67, Stmn1, Top2a, Tubb*
- Classical TIMs: *Ccl2, Ccl9, Ccr2, Cd14, Cd300lf, Cxcl10, F13a1, Fcn1, Fn1, Ifi205, Ifit2, Ifit3, Il1r2, Isg20, Itga4, Ly6c2, Lyz, Mgst1, Plaur, S100a8, S100a9, S100a12, Sell, Tgm2, Thbs1, Tlr2, Vcan*
- Nonclassical Monocytes: *Ace, Adgre4, Cd300a, Cdkn1c, Ceacam1, Ear2, Il17ra, Itgal, Lilrb2, Lrp1, Spn, Stk10, Tnfrsf1b, Treml4*
- M1 Macrophages: Orecchioni 2019, *In Vitro* Classically Activated
- M2 Macrophages: Orecchioni 2019, *In Vitro* Alternatively Activated

*Neutrophil-Related Gene Signatures* (23):

- T1: *Ltc4s, Mmp8, Mmp9, Ppia, Prr13, Ptma, Retnlg*
- T2: *Cxcr2, Cd300ld, Dusp1, Gbp2, Ifitm1, Il1b, Isg15, Jaml, Junb, Msrb1, Osm, S100a6, Selplg, Slpi*
- T3: *Atf3, Ccl3, Ccl4, Cd274, Cstb, Cxcl3, Hcar2, Hilpda, Hk2, Hmox1, Ier3, Jun, Ldha, Mif, Plin2, Spp1, Tgif1, Tnfrsf23, Vegfa, Zeb2*
- Mature: *Retnlg, Ccl6, S100a6, Clec4d, Prr13, Cebpb, Slpi, S100a11, Btg1, Cxcr2, Fth1, Grina, Mmp8, Fxyd5, Msrb1, H2-D1, Gm5483, Anxa2, Mmp9, Ftl1, Map1lc3b, Tmcc1, Sat1, Cyp4f18, Junb, Mxd1, Stk17b, Ypel3, Selplg, Il1f9, Dusp1, Slc16a3, Ccr1, Ifitm1, Rdh12, Clec4e, Arg2, Cd300ld, Amica1, Ctsd, Gda, Hacd4, Timp2, Fpr1, Ifi27l2a, Slc7a11, Stfa2l1, Il1b, Asprv1, Cxcl2*

### Cluster similarity analysis

To evaluate transcriptional congruence across immune cell clusters derived from distinct tumor models, we implemented a logistic regression model with elastic net regularization, as described by Cheng et al. (24). Clusters were downsampled to mitigate bias from unequal cell numbers. Cross-validation was used for model fitting, and predicted logits from test datasets were averaged and converted to probabilities to assess cluster similarity.

### Human scRNA-seq data collection and processing

To comprehensively profile human TIMEs, we leveraged the TISCH2 (25) public scRNA-seq database, which provides uniformly processed datasets via the MAESTRO workflow (26). We curated treatment-naïve primary tumor samples (Source/Tissue = Tumor) from breast invasive carcinoma (BRCA), colorectal cancer (CRC), and non-small cell lung cancer (NSCLC), restricting inclusion to studies with ≥2 patients and datasets with ≥10,000 cells, and encompassing both CD45+-enriched and unsorted preparations. All datasets were generated using 10x Genomics chemistry, with the exception of two that employed the inDrop platform (Azizi et al., 2018; Zilionis et al., 2019). We restricted analysis to immune-lineage cells (Celltype_malignancy = immune cells). Samples contributing fewer than 50 immune cells were excluded to mitigate low-yield noise. Study metadata were manually curated against the original publications, and cells with inconsistent lineage labels between TISCH2 and the source papers were removed when author-provided labels were available. To mirror the compartments analyzed in the syngeneic models, we extracted T cells, NK cells, dendritic cells, monocytes/macrophages, neutrophils, and mast cells from each human dataset. After filtering, 2,252,095 immune cells from 14 studies were retained and subsequently subjected to cross-study integration, unsupervised clustering, and systematic re-annotation using the same methods and criteria applied to the syngeneic datasets.

### Mouse–human comparisons

We compared cell states across species by restricting analyses to high-confidence one-to-one mouse–human orthologous gene pairs downloaded from the MGI database. Unsupervised cross-species comparisons were performed by hierarchical clustering of genes (rows) and cell states (columns) independently, using Ward’s linkage on Pearson correlation distance. For each species, we generated pseudobulk expression profiles for each annotated cell state by averaging single-cell expression values across all cells assigned to that state (Seurat AverageExpression). To mitigate dataset-specific effects, pseudobulk profiles were normalized within each dataset by scaling each profile to the median expression level across genes. Human and mouse pseudobulk matrices were then concatenated on the intersecting set of one-to-one orthologs and log transformed. For clustering, we restricted the gene set to positive markers for the focal cell population relative to other cell populations within each species, identified using Seurat FindMarkers (Wilcoxon rank-sum test). Genes were required to meet all of the following criteria: detected in at least 30% of cells in the focal population, Benjamini-Hochberg-adjusted *p*-value < 0.05, and log2 fold change ≥ 0.5.

To evaluate cross-species correlations in gene expression patterns for clusters of interest, we used marker genes from differential expression analyses performed separately in each species (Seurat FindMarkers). Differential expression analyses were conducted between the following groups: (i) Treg cells (human: Treg; mouse: CD4+T-C13) versus other T cells; (ii) CD8+ Tex cells (human: CD8_Tex_GZMB; mouse: aggregated CD8+T-C03, CD8+T-C05, and CD8+T-C06) versus other T cells; (iii) Mφ-SPP1 (human: aggregated Mφ_SPP1 and Mφ_PPARG; mouse: Mφ-C10) versus other Mo/Mφs; and (iv) Mo-ISG (human: Macro_C4; mouse: Mo-C04) versus other Mo/Mφs. Marker genes were defined as those detected in at least 5% of cells in the focal population, with log2 fold change ≥ 1, and Benjamini-Hochberg-adjusted *p*-value < 0.05. For each contrast, we intersected the human and mouse marker genes using the one-to-one ortholog map.

### Immunohistochemistry analysis

Five tumor samples from each model were harvested and fixed in 10% neutral buffered formalin for 24 hours, followed by embedding in paraffin. The paraffin blocks were sectioned into 4 μm slices and subjected to deparaffinization. Antigen retrieval was performed by immersing the slides in citrate buffer, followed by microwave treatment at high power for 2.5 minutes and incubation in a water bath at 95 °C for 30 minutes. Endogenous peroxidase activity was quenched by treating the slides with 3% hydrogen peroxide (H2O2) for 10 minutes at room temperature. The sections were then blocked with 10% goat serum for 1 hour. Post-blocking, the sections were incubated with anti-CD45 antibody (Cell Signaling Technology, Danvers, MA, USA, Cat# 70257S, 1:200) in a humidified chamber at 4 °C overnight. Following PBS washes, the slides were incubated with the secondary antibody (Cell Signaling Technology, Danvers, MA, USA, Cat# 8114L) for 1 hour at room temperature. Visualization was achieved using diaminobenzidine (DAB) as the chromogen. The slides were counterstained with hematoxylin and scanned using a Leica AT2 scanner.

Digital whole slide images were uploaded to the HALO Image Analysis platform (Indica Labs). Image analysis algorithms were developed using the Indica Labs Multiplex IHC module to detect CD45-positive cells by setting a positive threshold. The accuracy of the algorithms was validated through visual inspection by at least one pathologist. High infiltration was defined as CD45 positivity greater than 30%. A pathologist reviewed the slides to evaluate the differentiation status of the tumor (**Supplementary Table 1****).**

### Somatic mutations and tumor mutational burden

Somatic mutations in syngeneic models were called from cell line whole-exome sequencing using Sentieon in tumor-only mode. For downstream analyses, we retained only nonsynonymous variants (missense, nonsense, and frameshift indels) that did not overlap dbSNP entries for the corresponding genetic background. Genes that are common drivers in human cancers were reported as representative examples. Tumor mutational burden (TMB) was calculated as the number of mutations per megabase (Mb), considering variants with a variant allele frequency greater than 1% (**Supplementary Table 1**).

### Bulk RNA-seq and immune deconvolution

RNA-seq libraries were prepared using NEBNext^®^ UltraTM RNA Library Prep Kit for Illumina^®^ (NEB, USA), following the manufacturer’s protocol. Libraries quality was assessed prior to sequencing on the Illumina NovaSeq 6000 platform. Sequencing read quality was assessed using FastQC. Reads were aligned to the mouse reference genome (mm10, Ensembl release 98) using STAR (v.2.5. 4b), and gene-level quantification was performed with RSEM (v.1.3.1). Normalized expression data were subjected to immune cell deconvolution using the quanTIseq (27) algorithm, with all immune-related components aggregated under the “quanTIseq_immune” category.

### Statistical analysis

Tumor volume differences among groups were analyzed using one-way analysis of variance (ANOVA) in GraphPad Prism (version 10.1.2), with groupings defined by a single independent variable. Data are presented as mean ± standard error of the mean (SEM) from independent experiments. Statistical significance was set at *p* ≤ 0.05. Models were classified as anti-PD-1 responsive if the treatment group exhibited a statistically significant reduction in tumor volume compared to the control group at the study endpoint; otherwise, they were considered anti-PD-1 resistant.

### Data availability

The data generated in this study are available at Gene Expression Omnibus (GEO) accession no. GSE307143.

## Results

### Immune cell composition landscapes across ten syngeneic tumor models

**Figure 1:**
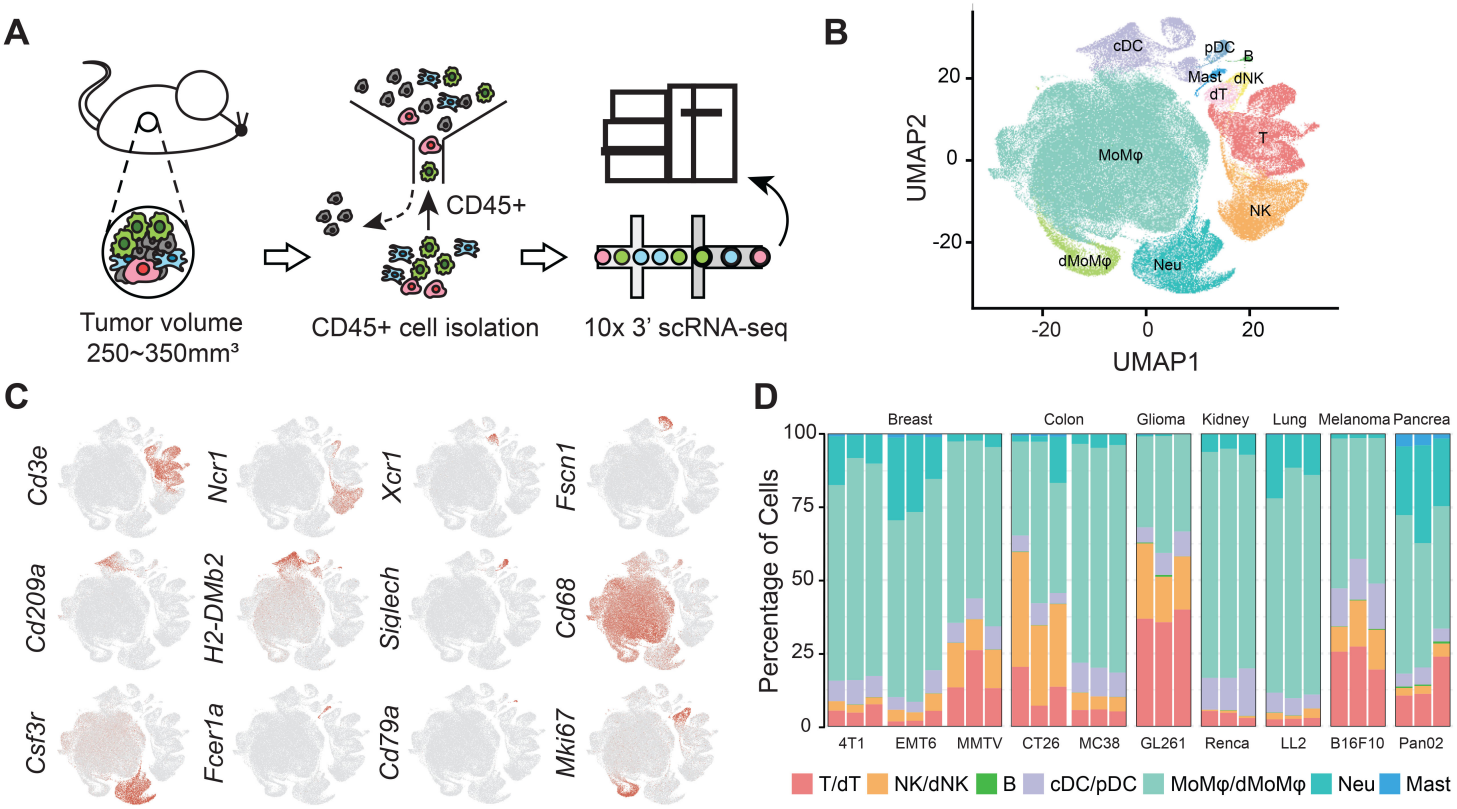
High-resolution profiling of TIICs across murine syngeneic tumor models. **(A)** Schematic representation of the experimental workflow for isolating and processing Cd45+ immune cells from 10 murine tumor models: breast mammary carcinoma (4T1, EMT6, MMTV-PyMT), colon carcinoma (CT26.WT, MC38), glioma (GL261), renal adenocarcinoma (Renca), lung carcinoma (LL2), melanoma (B16F10), and pancreatic adenocarcinoma (Pan02), with three biological replicates per model. **(B)** UMAP visualization of CCA integrated data depicting 7 major immune cell populations. **(C)** Expression patterns of canonical marker genes utilized to identify lymphoid (T cells, NK cells, B cells) and myeloid (DCs, Mo/Mφs, neutrophils, mast cells) lineages. **(D)** Percentages of major immune cell types in TIICs for each model.

To elucidate the high-resolution landscapes of tumor-infiltrating immune cells (TIICs) within murine syngeneic tumor models, we performed scRNA-seq on CD45+ immune cells isolated from ten treatment-naïve models, encompassing seven prevalent cancer types: breast mammary carcinoma (4T1, EMT6, MMTV-PyMT), colon carcinoma (CT26.WT, MC38), glioma (GL261), renal adenocarcinoma (Renca), lung carcinoma (LL2), melanoma (B16F10), and pancreatic adenocarcinoma (Pan02) (**Figures 1A, D**). After stringent quality control, 166,861 immune cells were retained, with a median of 3,096 genes and 12,705 unique molecular identifiers (UMIs) per cell (**Supplementary Figures S1A, B**). Unsupervised clustering revealed seven major immune cell populations, each distinguished by the expression of canonical markers: T cells, natural killer (NK) cells, and B cells, monocytes/macrophages (Mo/Mφs), neutrophils, dendritic cells (DCs), and mast cells (**Figures 1B, C**, **Supplementary Figure S1E**, **Supplementary Table 2**).

Mo/Mφs constituted the dominant immune population, accounting for 60.58% of TIICs across models, with proportions ranging from 34.61% in GL261 to 76.49% in Renca. Notably, neutrophils were significantly represented in our dataset, comprising 9.66% of TIICs, with their presence varying from 0.41% in GL261 to 26.90% in Pan02. The distribution of lymphoid cells also exhibited considerable variability, with T cells accounting for 11.99% and NK cells comprising 9.10% of TIICs. Specifically, GL261 and CT26.WT exhibited the highest proportions of T and NK cells in their TIICs at 57.60% and 45.56%, respectively. Conversely, LL2 and Renca presented the lowest proportions, at 4.75% and 4.91%, respectively (**Figure 1D**).

### Transcriptomic diversity among T cell populations

**Figure 2:**
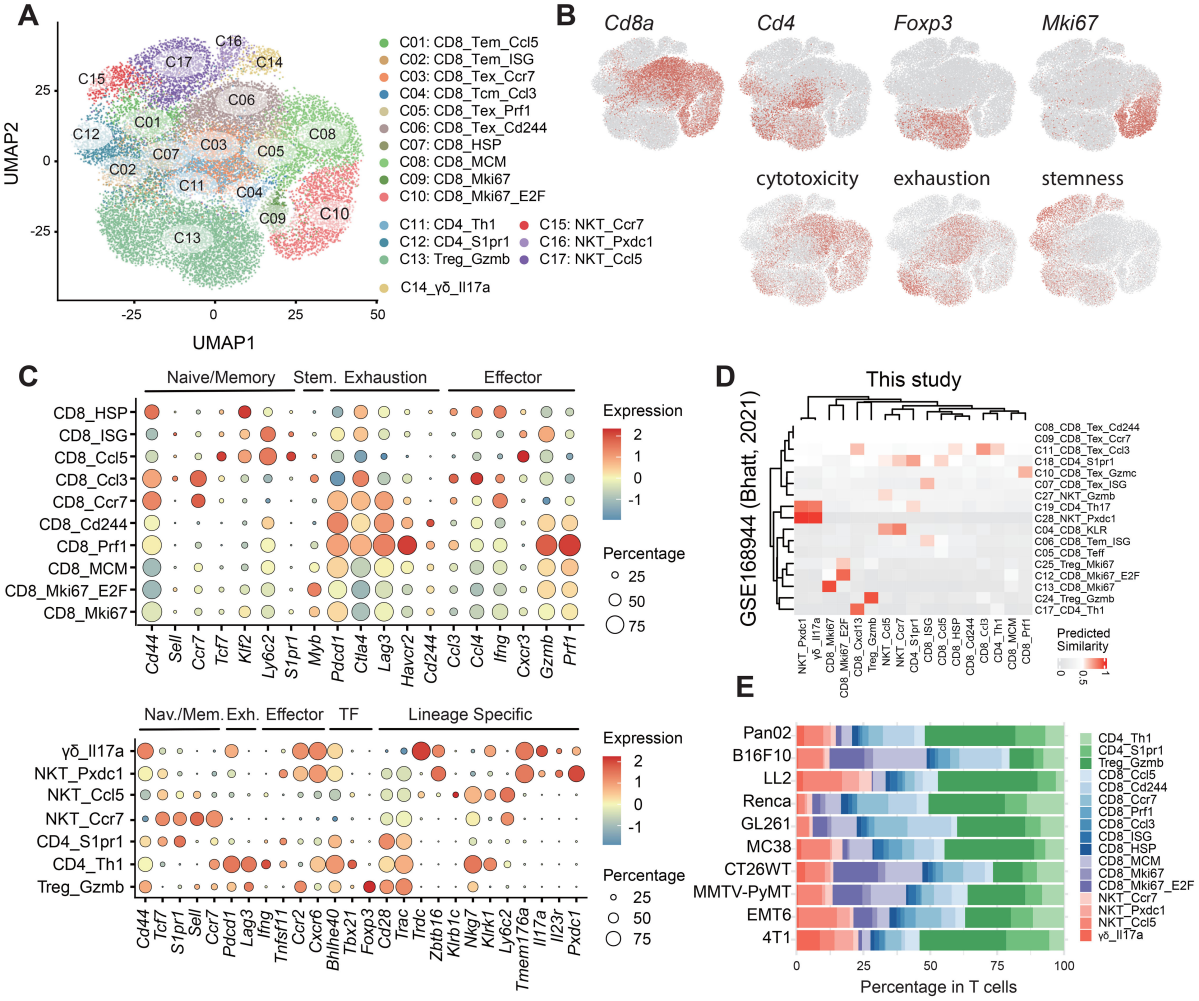
Identification and characterization of T cell subpopulations. **(A)** UMAP projection illustrating 17 distinct T cell subpopulations: 10 CD8+ subsets (C01-C10), 3 CD4+ subsets (C11-C13), and 4 double-negative (CD8-CD4-) subsets (C14-C17). **(B)** Expression profiles of canonical markers *Cd8a*, *Cd4*, *Foxp3* and *Mki67*, along with gene signatures associated with cytotoxicity, exhaustion, and stemness. **(C)** Transcriptomic characterization of CD8+ (top) and CD4+ (bottom) T cells, revealing effector memory, central memory, and exhausted phenotypes in CD8+ T cells, and Th1 helper, EM-like, and Treg phenotypes in CD4+ T cells; dot color and size represent the proportion of expressing cells and average expression level, respectively. **(D)** Comparative analysis of T cells showing the similarity between the transcriptional profiles obtained in this study and those documented in the published mouse model data (GSE168944). **(E)** Proportional distribution of each T subset within the total T cell compartment.

T cells are pivotal in orchestrating antitumor immunity. To elucidate the intricate role of T cells within syngeneic tumors, we identified seventeen distinct T cell subpopulations, comprising ten CD8+ subsets (C01-C10), three CD4+ subsets (C11-C13), and four double-negative (CD8-CD4-) subsets (C14-C17). Each subset exhibited unique signatures (17) associated with cytotoxicity, exhaustion, and stemness, alongside the expression of key marker genes (**Figures 2A–C**, **Supplementary Table 3**). Our dataset demonstrated a high degree of consistency with T cells in the MC38 syngeneic model that were previous extensively characterized (9) in terms of both component abundance and transcriptional profiles (**Figure 2D**, **Supplementary Figure S2**).

The CD8+ compartment comprised two effector memory (EM; C01, C02), one central memory-like (CM; C04), and three exhausted (EX; C03, C05, C06) subsets (**Figures 2A, C** upper panel). T-EM cells were characterized by elevated expression of *Tcf7*, *Klf2*, and *S1pr1*, along with EM-like predicted markers *Ly6c2* and *Cxcr3* (17). Cluster C02 exhibited further enrichment for interferon-stimulated genes (ISGs; *Ifit1*/*3*, *Isg15*). T-CM cells were distinguished by the presence of canonical CM cell markers (*Cd44*, *Sell*, *Ccr7*), chemokines (*Ccl3*, *Ccl4*, *Xcl1*), and *Myb*, which signifies their stemness status (28). T-EX cells exhibited increased expression of genes (*Pdcd1*, *Havcr2*, *Ctla4*, *Lag3*, *Havcr2*) and signatures associated with exhaustion. Among them, cluster C03 exhibited lower expression of *Havcr2* and *Cd244*, reduced levels of effector molecules (*Gzmb*, *Prf1*) and increased expression of *Ccr7*, indicating a less exhausted or pre-dysfunctional state. Cluster C05 expressed elevated levels of the CD8 T cell activation marker *Cx3cr1*, along with granzyme molecules (e.g. *Gzmb*) and *Prf1*, indicating potential effector function in action. Additionally, our dataset revealed one cluster (C07) displaying high levels of heat shock proteins (HSPs), including *Hspa1a*/*b* (Hsp72/70) and *Hsp90aa1*/*Hspab1* (Hsp90), as well as other markers indicative of cellular stress, such as *Dnajb1*/*Dnaja1* (29). Furthermore, numerous proliferative subpopulations (C08-C10) were identified based on the expression of MCM (*Mcm2*-*7*), *Mki67*, and *E2F*.

Within the CD4+ compartment (**Figures 2A, C** lower panel), we identified two conventional CD4+ T cell clusters (C11 and C12) and one regulatory T cell (Treg) cluster (C13). Cluster C11 exhibited a Th1-like transcriptional profile, marked by elevated expression of *Tbx21*, *Ifng*, and *Bhlhe40*, alongside exhaustion-associated genes such as *Pdcd1* and *Lag3*. Cluster C12 displayed high levels of *Tcf7*, *Lef1*, *S1pr1*, and *Il7r*, with low expression of *Ccr7* and *Sell*, indicative of an EM-like phenotype.

The CD8-CD4- compartment included one γδ T cell population (C14) and three NKT cell populations (C15-C17) (**Figures 2A, C** lower panel). Cluster C14 was defined by a prominent TCR γδ repertoire and elevated expression of *Il17a*. The 4T1 model exhibited a markedly higher proportion of γδ T cells compared to other models (**Figure 2E**). Clusters C15-C17 were classified as NKT cells based on co-expression of conventional T cell markers (*Cd3e*, *Cd28*), and NK cell-associated genes, including *Zbtb16* (PLZF), *Klrb1c* (NK1.1/Cd161), *Klrk1* (Nkg2d), and *Nkg7*. These cells also displayed a restricted TCRαβ repertoire. Cluster C15 exhibited high expression of *Tcf7*, *Lef1*, *Sell*, and *Ccr7* but low levels of *Cd44* and effector molecules, indicative of a naïve phenotype. Cluster C16, enriched for *Zbtb16* and Th17-associated genes (*Rorc* (RORγt), *Il17a*, *Il23r*, *Pxdc1*), was annotated as NKT17 cells. Cluster C17, which constituted the majority (69.3%, 2,974 cells) of CD8-CD4- T cells, expressed elevated levels of *Ccl5* and *Ly6c2*, exhibiting an EM-like phenotype.

### Diversification of NK cells

**Figure 3:**
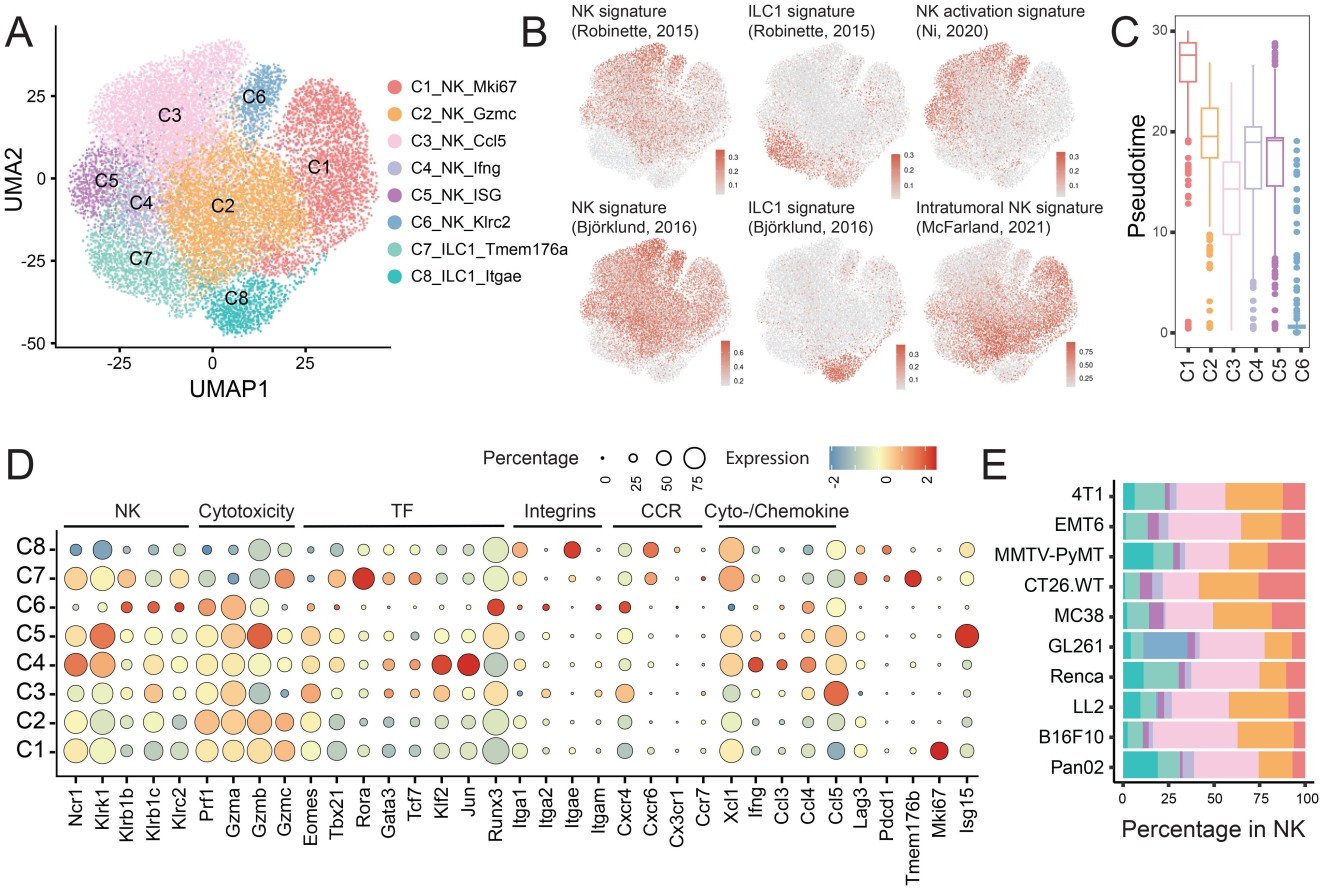
Characterization of intratumoral NK cell and ILC clusters. **(A)** UMAP plot showing NK and ILC subpopulations. **(B)** UMAP projection of signature scores for NK cells, ILCs, NK cell activation and intratumoral NK identity. **(C)** Pseudotime trajectory analysis illustrating the developmental progression of NK cell clusters (C1-C6). **(D)** Bubble heatmap displaying expression of genes linked to lineage, cytotoxicity, transcription factors (TFs), integrins, chemokine receptors (CCRs), and cytokines/chemokines across NK and ILC subsets. **(E)** Proportional distribution of NK and ILC subset within the total NK population (refer to **Figure 1D**).

To investigate the heterogeneity of intratumoral NK cells, we re-clustered *Ncr1+Cd3e-* cells, identifying six distinct NK cell subsets (C1-C6) and two group 1 innate lymphoid cell (ILC) subsets (C7-C8) (**Figure 3A**, **Supplementary Table 4**).

Clusters C1 and C2 were defined by high expression of perforin and granzyme, with C1 specifically identified as a proliferating NK cell population. Clusters C3-C5 exhibited distinct cytokine and chemokine profiles: C3 was enriched for *Ccl5*, C4 for *Ifng*, and C5 for ISGs (*Isg15*, *Ifit1*, *Ifi203*) (**Figure 3D**). Cluster C6 was notable for its elevated expression of *Klrc2* (encoding NKG2C) and *Klrb1b*/*c*, but reduced expression of *Ncr1* and *Klrk1* (encoding NKG2D) (**Figure 3D**). It also expressed major histocompatibility complex (MHC) class II molecules and associated genes (*H2-Aa*, *Cd74*), consistent with an adaptive NK phenotype (30). These cells exhibited a high NK activation signature score (20) (**Figure 3B**), and demonstrated the highest expression level of *Itga2* (Cd49b) while showing a low level of the intratumoral NK signature score (20), indicating a conventional NK cell phenotype (CD49b+) rather than a tissue-resident profile marked by *Itga1* (CD49a). Cluster C6 was predominantly enriched in the GL261 model (**Figure 3E**).

Consistently, cluster C6 was positioned at one end of the pseudotime trajectory (**Figure 3C**), followed by cluster C3, which exhibited moderate *Itga2* expression, restricted *Itga1* expression, a high NK activation signature score, and a low intratumoral NK signature. In contrast, two KLRG1+ NK cell populations, clusters C1 and C2, were positioned at the terminal intratumoral pseudotime state and accordingly displayed a diminished NK activation signature score alongside an elevated intratumoral NK signature score. This aligns with previous reports indicating that NK cells rapidly lose their effector functions within the TME (20).

The ILC1 clusters (C7 and C8) were defined by high expression of *Rora*, *Il7r* (CD127/IL-7Rα), and *Itga1*, absence of *Eomes*, and reduced levels of cytotoxic molecules, distinguishing them from bona fide NK cells. Their identities were further validated using established ILC1-specific signatures (18, 19) (**Figures 3B, D**).

### Distinct subsets of DCs

**Figure 4:**
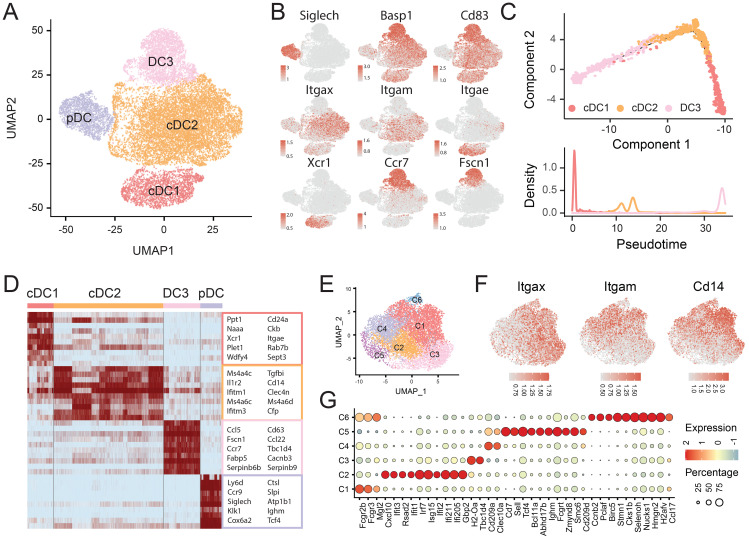
Transcriptional heterogeneity of DCs. **(A)** UMAP plot identifying 4 DC subtypes. **(B)** Expression of canonical DC markers overlaid on UMAP plots. **(C)** Developmental trajectory of cDC1, cDC2 and DC3 subsets inferred by Monocle2. **(D)** Heatmap showing the expression of selected differential expressed genes across clusters. **(E)** UMAP visualization of cDC2 subclusters. **(F)** UMAP plot showing the expression of *Itgax*, *Itgam*, and *Cd14* in cDC2 cells. **(G)** Bubble heatmap showing selected differential expressed genes across 6 cDC2 subclusters.

DCs within the TME are a heterogeneous population critical for initiating and modulating both innate and adaptive immune responses. Based on canonical markers and functional gene expression profiles, we classified DCs into four distinct subtypes: one plasmacytoid DC subset (pDC, marked by *Siglech*), two conventional DC subsets (cDC1 and cDC2, marked by *Itgax* (CD11c)), and one *Ccr7*+ DC subset (DC3) (**Figures 4A, B, D**, **Supplementary Table 5**).

pDCs, typically associated with type I interferon production and antitumor activity, exhibited elevated *Lag3* and *Cd37* expression, corroborating previous findings (31) that suggests their inhibitor roles in modulating the local immune response. The cDC1 subset, defined by *Xcr1* and *Clec9a*, specializes in antigen cross-presentation and activation of cytotoxic CD8+ T cells. This population includes both CD8a+ and CD103+ (encoded by *Itgae*) cells, with CD103+ cDC1s predominating in our dataset (**Figures 4B, D**).

The cDC2 subset, marked by *Itgam* (CD11b) and *Sirpa* (CD172a), also expressed high levels of *Cd14*, identifying them as *Itgax*+*Itgam*+*Cd14*+ monocyte-derived dendritic cells (MoDCs) (**Figure 4F**). Unlike cDC1, cDC2 primarily activate CD4+ T helper cells. Transcriptome analysis revealed substantial heterogeneity within cDC2s, with two peaks in pseudotime trajectories (**Figure 4C**) and multiple compartments in the marker gene heatmap (**Figure 4D**). We further resolved cDC2s into six subclusters: C1 (*Mgl2* (CD301b)), C2 (ISGs), C3 (*Tbc1d4*), C4 (*Clec10a*), C5 (*Selp*, *Abhd17b*, *Fcgrt*), and C6 (*Ccnb2*, *Birc5*, *Stmn1* (proliferation/differentiation)) (**Figures 4E–G**).

The DC3 subset was identified by its elevated expression of *Ccr7*, *Cd83*, and *Fscn1*, despite lacking conventional DC markers. Both *Ccr7* and *Cd83* are well-established indicators of DC maturation, while *Fscn1* supports migratory capacity toward lymph nodes (32) (**Figures 4B, D**).

### Functional characterization of Mo/Mφ populations

**Figure 5:**
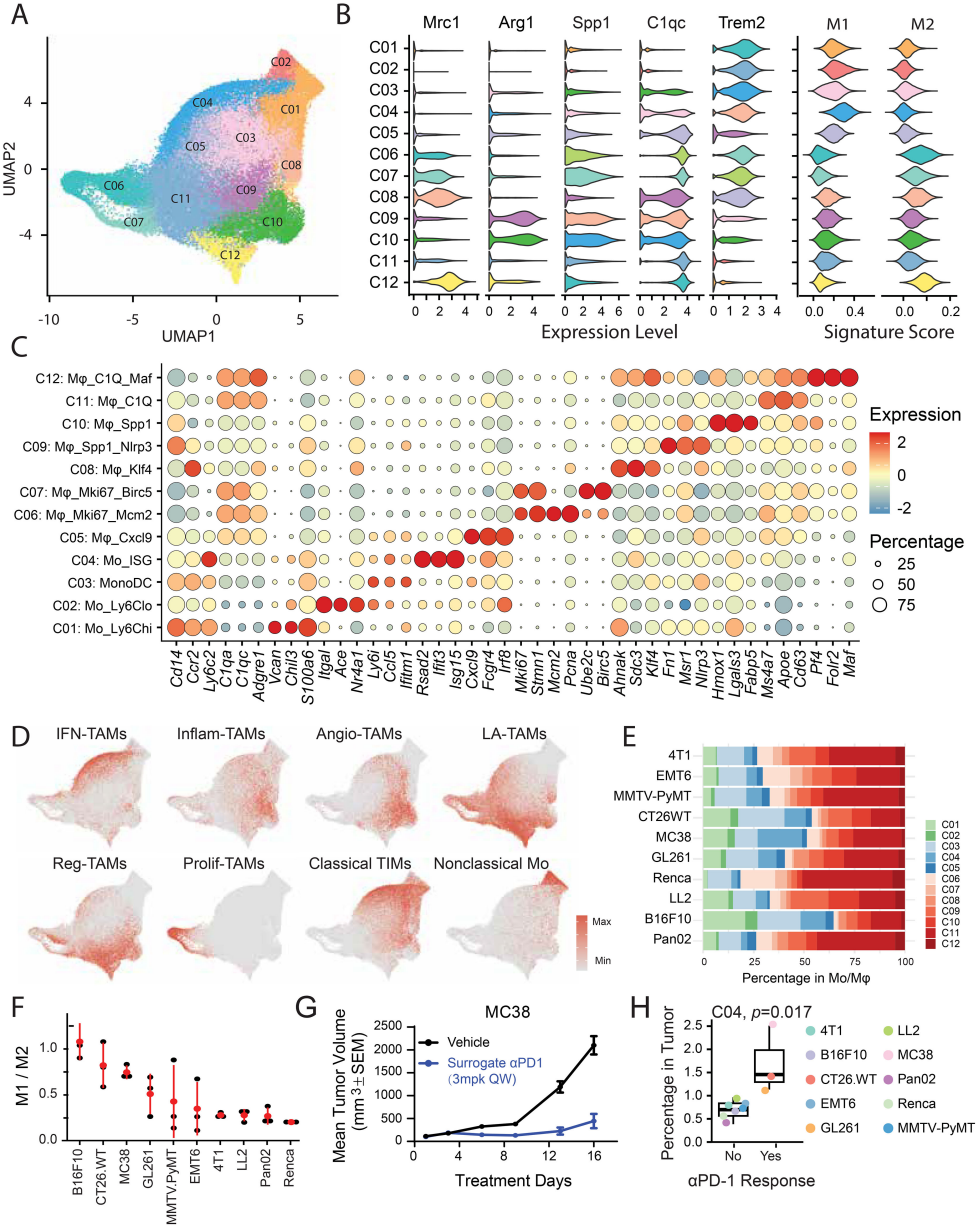
Molecular and functional diversity of tumor-infiltrating Mo/Mφs. **(A)** UMAP visualization of distinct Mo/Mφ clusters within the TME. **(B)** Violin plots showing expression of Mo/Mφ markers, along with M1- and M2-associated gene signatures across clusters. **(C)** Bubble heatmap of representative signature genes across 12 Mo/Mφ subsets. **(D)** Expression of signature gene sets superimposed on the UMAP plot. **(E)** Proportional representation of each Mo/Mφ subset within the total Mo/Mφ population. **(F)** Ranked M1/M2 ratio across models based on established marker genes, indicating Mo/Mφ polarization states. **(G)** Evaluation of anti-PD-1 treatment efficacy in MC38 tumor models. **(H)** Bar plots comparing the abundance of the cluster C04 between anti-PD-1–responsive and –resistant tumor models.

Tumor-associated macrophages (TAMs) are key regulators of tumor progression and represent promising targets for immunotherapy. Given their abundance, we conducted a detailed transcriptional and functional analysis, identifying twelve distinct subclusters: three monocyte subsets (Mo, C01-C02, C04), one monocyte and DC subset (MonoDC, C03), and eight macrophage subsets (Mφ, C05-C12). By aligning these subpopulations with canonical M1 (proinflammatory/antitumor) and M2 (anti-inflammatory/protumor) phenotypes (22), we determined that clusters C01-C05 displayed M1-like characteristics based on their expression of M1-associated markers and signatures. Conversely, clusters C06-C12 were classified as M2-like due to their upregulation of M2-associated markers and signatures (**Figures 5A–C**, **Supplementary Table 6**).

Clusters C01 and C02 exhibited the lowest expression of *Adgre1* (F4/80), *Apoe*, and *C1qa*/*c*, and were classified as Ly6Chi and Ly6Clo monocytes, respectively, based on differential expression of *Ly6c2*, *Ccr2*, *Cd14*, and *Vcan* (C01) and *Nr4a1*, *Ace*, and *Itgal* (C02). Cluster C03 represented the MonoDC subset, characterized by high expression of DC-associated genes, including MHC class II molecules and *Cd74*. Cluster C04, marked by elevated expression of ISGs, showed moderate expression of macrophage markers and high levels of *Ly6c2* and *Tgfbi*, suggesting an intermediate state between monocytes and macrophages.

Among macrophage subsets, C05 was the only M1-like population, distinguished by high *Cxcl9* expression and linked to favorable responses to anti-PD-L1 therapy (11). Clusters C06 and C07 represented proliferative macrophages, while C08 showed increased expression of *Ccr2* and *Klf4*. Clusters C09-C12 displayed transcriptional profiles resembling previously described SPP1+ and C1Q+ TAMs (10, 24). Specifically, C09 and C10 upregulated *Spp1* and *Arg1*, while C11 and C12 exhibited high levels of C1Q complement components. C12 also expressed *Pf4*, *Folr2*, *Crb2*, *Cd163*, and *Lyve1*, markers characteristic of tissue-resident macrophages.

It is important to note that the M1 and M2 gene signatures are not entirely mutually exclusive, as the M1/M2 dichotomy was established in the pregenomic era based largely on *in vitro* stimulation studies with type 1 or type 2 cytokines. To better capture the functional and molecular diversity of tumor-infiltrated Mo/Mφs, we applied a nomenclature informed by single-cell omics data (21) (**Figure 5D**). Consistent with marker-based annotation, clusters C01 and C04 exhibited strong classical tumor-infiltrating monocyte (TIM) signature, while C02 aligned with a nonclassical monocyte signature. C04 also showed the highest signature score of interferon-primed TAMs (IFN-TAMs). Among macrophage subsets, C09 and C10 were enriched for inflammatory cytokine-enriched TAMs (Inflam-TAMs), whereas C12 showed the strongest lipid-associated TAMs (LA-TAMs) signature, linked to immunosuppressive and tolerance-related functions.

To assess the role of Mo/Mφ subsets in immunotherapy response, we quantified the M1/M2 ratio across models. Notably, MC38 tumors, despite low T and NK cell infiltration, harbored abundant Mo/Mφs with a relatively high M1/M2 ratio (**Figures 1D**, **5E, F**), potentially explaining their sensitivity to anti-PD-1 therapy (**Figure 5G**). To further investigate the involvement of Mo/Mφ subsets in anti-PD-1 response, we assessed their enrichment in responsive tumor models. Initial efficacy testing across syngeneic models identified MC38, GL261, and CT26 as responsive to anti-PD-1 therapy, while others were resistant (**Supplementary Figure S3**). We then estimated the absolute abundance of Mo/Mφ subpopulation by integrating their proportions within TIICs and the overall immune cell content in tumors, inferred from bulk RNA-seq via deconvolution (**Supplementary Figures S4A–C**). Stratification by anti-PD-1 sensitivity revealed that Cluster C04 (ISG^high^ monocytes) was significantly enriched in responsive models (**Figure 5H**, **Supplementary Figure S4D**). Notably, a recent study demonstrated that inflammatory/ISG-enriched monocytes promoted the expansion of tumor-specific CD8+ T cells and amplify antitumor immunity; transcriptionally, these monocytes were highly congruent with our ISG^high^ subset (33).

### Multifaceted roles of neutrophils in the TME

**Figure 6:**
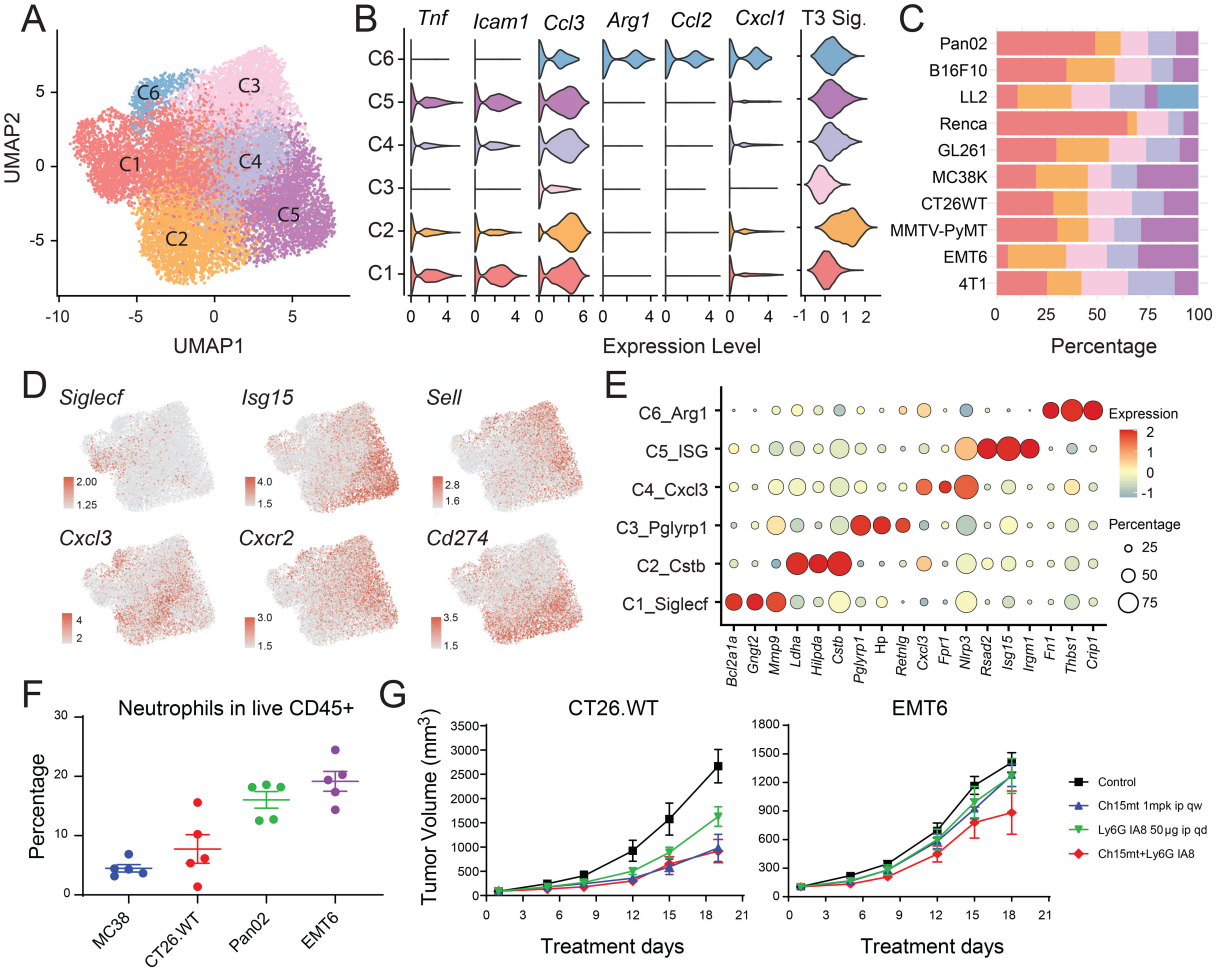
Transcriptomic diversity of tumor-infiltrating neutrophils. **(A)** UMAP plot illustrating the landscape of tumor-infiltrating neutrophil subtypes. **(B)** Violin plot showing expression of canonical N1 and N2 markers, along with terminal differentiation signatures across neutrophil subclusters. **(C)** Proportional distribution of neutrophil subclusters within the total neutrophil population across different tumor models. **(D)** UMAP plots displaying expression of key functional genes. **(E)** Bubble heatmap of selected differentially expressed genes across neutrophil subclusters. **(F)** Flow cytometry validation of neutrophil abundance in four tumor models. **(G)** Effect of Ly6G-mediated neutrophil depletion on tumor growth in MC38 and EMT6 models, alone or in combination with anti-PD-1 therapy.

Neutrophils are often underrepresented in scRNA-seq studies due to their short lifespan and low RNA content. A considerable population of neutrophils was detected across various models in our dataset, as corroborated by flow cytometric analysis (CD11b+CD115-Ly6G+) (**Figure 6F**), in close concordance with the proportions depicted in **Figure 1E**. We delineated six distinct neutrophil subsets, each defined by discrete gene expression profiles and functional attributes. Cluster C1 was enriched for *SiglecF*, while Clusters C2, C4, and C6 exhibited elevated levels of *Cxcl3*. Clusters C3 and C5 were distinguished by high levels of *Sell*. *SiglecF*-high and *Cxcl3*-high neutrophils were previously reported exclusively in tumor-bearing tissues (34, 35) (**Figures 6A-E**, **Supplementary Table 7**).

Analogous to the M1/M2 paradigm in macrophages, tumor-associated neutrophils (TANs) adopt either N1 (antitumor) or N2 (protumor) phenotypes (36). Cluster C6 displayed transcriptional features consistent with an N2-like state, including upregulation of *Arg1*, *Ccl2*, and *Cxcl1*, as well as genes related to angiogenesis such as *Thbs1* and *Lagls3* (**Figure 6B**), suggesting a pronounced pro-tumoral role. This subset was predominantly observed in LL2 models (**Figure 6C**). Cluster C1, characterized by high *SiglecF* expression, has also been implicated in protumor activities. Cluster C2, marked by elevated *Cstb* and *Ccl3*, closely resembled the mN5 populations described by Zilionis et al. (34), and exhibited the highest T3 signature score (23), indicating a terminally differentiated, pro-tumor neutrophil subset (**Figure 6B**). Cluster C3 had the highest neutrophil maturation score and was significantly associated with migration (**Supplementary Figures S5A, B**), suggesting it represented newly infiltrating mature neutrophils. In contract, cluster C5 expressed high levels of type I ISGs (*Ifit1*, *Irf1*, *Rsad2*, *Isg15*, and *Cxcl10*), aligning with the mN2 subset known to expand during effective immunotherapy (e.g., anti-PD-1 treatment) (34, 35, 37) (**Supplementary Figure S5C**).

To further investigate the functional relevance of TANs, we performed *in vivo* depletion experiments using anti-Ly6G antibodies (**Figure 6G**; **Supplementary Figure S5D**). Consistent with previous studies (38), neutrophil depletion significantly reduced tumor burden in CT26.WT models, whereas EMT6 tumors remained unresponsive. Notably, neutrophil depletion failed to enhance the efficacy of PD-1 blockade in both CT26.WT (anti-PD-1 responsive) and EMT6 (anti-PD-1 non-responsive) models (**Figure 6G**). Comparative profiling of neutrophil subsets and effector molecule expression across CT26.WT and EMT6 revealed that, although EMT6 harbored a greater total neutrophil burden (**Figure 1D**), it contained a significantly lower proportion of the C1/SiglecF^high^ subset, only modest, non-significant increase in C2 and C5, and reduced expression of protumor mediators such as *Arg1* and *Tgfb1*, relative to CT26.WT (**Supplementary Figures S5E, F**). These features indicated that CT26.WT exhibited a more protumor-skewed neutrophil program, rendering neutrophil depletion measurably efficacious, whereas in EMT6, where neutrophils were comparatively less protumor, depletion conferred limited benefit. The ablation of the C5/ISG^high^ subset in both models may therefore underlie the lack of synergy between neutrophil depletion and PD-1 blockade (35, 37). Together, these findings underscored the phenotypic diversity and functional complexity of neutrophils within the TME, highlighting their multifaceted roles in modulating antitumor immunity and therapeutic response.

### Comparison of human and mouse TIME

To assess the translational relevance of TIICs across species, we integrated 14 treatment-naive human scRNA-seq datasets spanning BRCA, CRC, and SCLC, processed with a uniform pipeline (Materials and Methods, **Table 3**).

**Table 3:** Summary of dataset sources, patient numbers, and cell counts.

Cancer Type	Dataset Name	Patients	Cells	PMID
BRCA	Azizi, Cell, 2018	8	7,413	29961579
	Qian, Cell Res, 2020	14	18,525	32561858
	Gao, Nat Biotechnol, 2021	5	3,049	33462507
	Pal, EMBO J, 2021	31	46,978	33950524
	Wu, Nat Genet, 2021	20	3,0052	34493872
CRC	Qian, Cell Res, 2020	7	12,075	32561858
	Wu, Nature, 2020	2	6,862	32103181
	Zhang, Cell, 2020	9	10,693	32302573
	Uhlitz, EMBO Mol Med, 2021	12	21,636	34409732
NSCLC	Lambrechts, Nat Med, 2018	5	21,543	29988129
	Song, Cancer Med, 2019	4	2,906	31033233
	Zilionis, Immunity, 2019	6	17,737	30979687
	Kim, Nat Commun, 2020	7	19,750	32385277
	Wu, Nature, 2020	6	32,876	32103181

**Figure 7:**
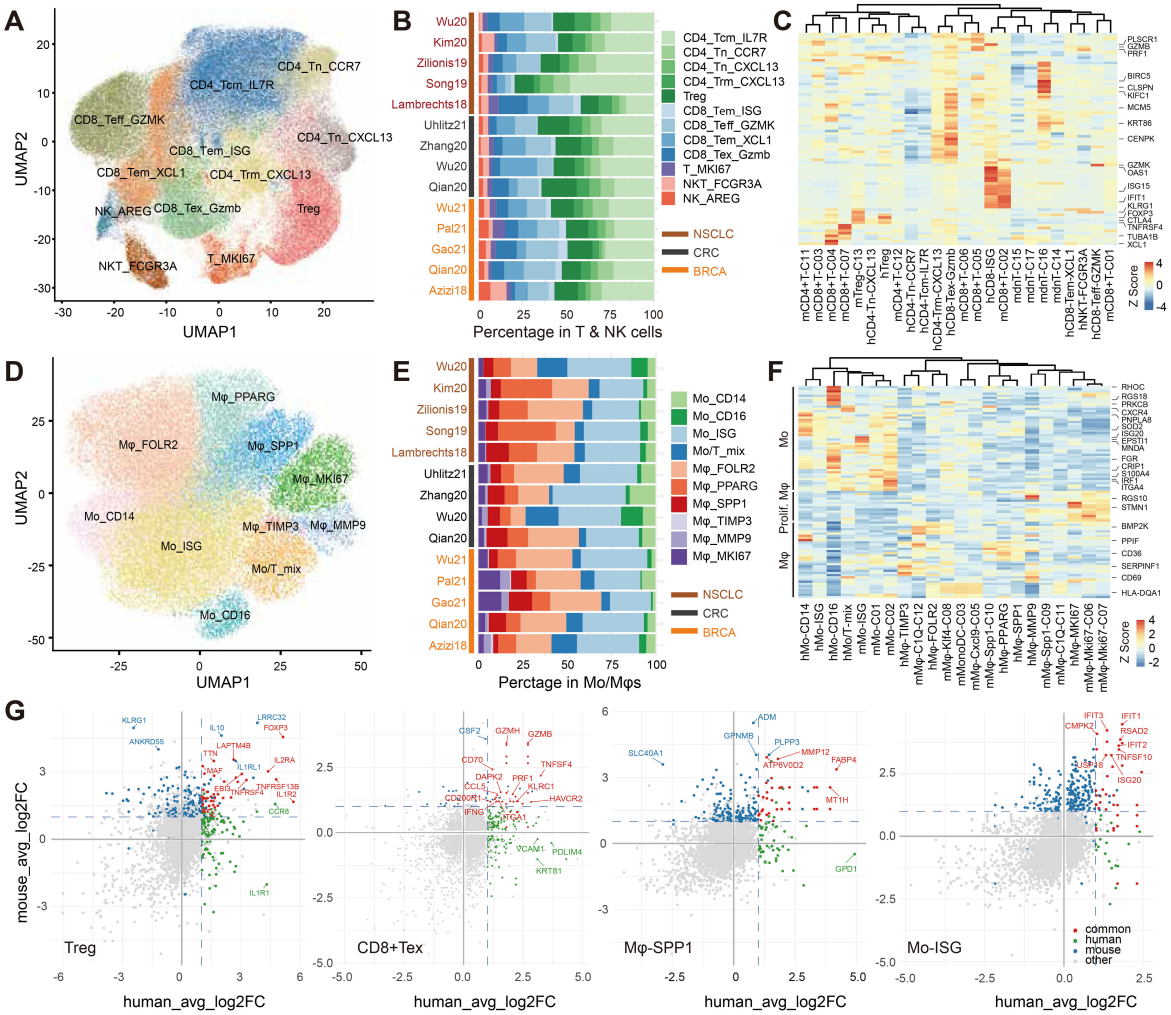
Cross-species profiling of intratumoral immune subsets in public human datasets and syngeneic tumors in this study. **(A, D)** UMAP embeddings of T/NK cells **(A)** and Mo/Mφ **(D)**, annotated into transcriptional subpopulations using canonical markers after batch integration. **(B, E)** Compositional profiles of T/NK **(B)** and Mo/Mφ **(E)** compartments, shown as proportions within each corresponding parent compartment and summarized per study. **(C, F)** Heatmaps of one-to-one orthologs with concordant enrichment in human and mouse T cell **(C)** and Mo/Mφ **(F)** states, scaled by row Z-score. **(G)** Correlation of marker genes across Treg, CD8+ Tex, Mφ-SPP1, and Mo-ISG clusters in human and murine syngeneic datasets. For each species, gene-level fold changes versus other clusters are plotted. Shared significant markers are highlighted in red; mouse-specific in blue; human-specific in green.

Within human tumors, T cells constituted a majority of TIICs (58.3% overall; BRCA 53.0%, CRC 47.3%, NSCLC 68.4%), exceeding the proportional T-cell representation in syngeneic mouse models. We resolved four CD8+ subsets (CD8_Teff_GZMK, CD8_Tem_XCL1, CD8_Tex_GZMB, CD8_ISG), four conventional CD4+ subsets (CD4_Tn_CCR7, CD4_Tn_CXCL13, CD4_Tcm_IL7R, CD4_Trm_CXCL13), one Treg subset, one proliferating subset (T_MKI67), and one NKT subset (**Figure 7A**), supported by canonical memory/exhaustion markers and distinct transcriptional programs (**Supplementary Figure S6A**). In human tumors, Treg cells comprised 22.9% of CD4+ T cells and exhausted CD8+ T cells 21.9% of CD8+ T cells; proliferating T cells and NKT cells represented 3.6% and 3.3% of total T cells, respectively. In contrast, syngeneic models displayed elevated frequencies of exhausted CD8+ T cells (40.8% of CD8+), Treg cells (60.4% of CD4+), proliferating T cells (20.6% of total T cells across CD8_MCM and CD8_Mki67 clusters), and NKT cells (11.1% across three clusters; **Figure 7B**).

At the level of major T-cell lineages (CD8+, CD4+, Treg, NKT), cell states were conserved across species, as evidenced by concordant expression of orthologous marker genes (**Supplementary Figure S6B**). By contrast, integrative profiling of subtype-discriminating markers in human and mouse T cells revealed only modest concordance of cell states across species, with subpopulations partitioning primarily by species rather than by presumed functional equivalence (**Figure 7C**). However, differential expression analyses focusing on Treg and exhausted CD8+ T cells identified shared transcriptional features across species. In Tregs, *FOXP3* and *IL2RA* showed robust conservation, with additional overlap in *TNFRSF4*, *IL1R2*, and *EBI3*. Notably, some genes demonstrated opposing patterns: *IL1RA* was enriched in human Tregs but diminished in mouse Tregs, whereas *KLRG1* was elevated in mouse Tregs but low in humans. In exhausted CD8+ T cells, conserved markers included *HAVCR2*, *TNFSF4*, and cytotoxic effectors from the granzyme and perforin families, supporting a shared exhaustion/cytotoxic module. Together, these findings indicated that despite fine-grained T-cell subsets exhibited only modest conservation of cellular states across species, therapeutically relevant Tregs and CD8+ Tex cells displayed conserved marker profiles and preserved functional programs (**Figure 7G**, **Supplementary Table 14**).

Myeloid lineages likewise displayed conserved cell states across species, both at the level of major lineages and within Mo/Mφ sublineages (**Supplementary Figure S6B**, **Figure 7F**). In human tumors, Mo/Mφ cells constituted 18.4% of TIICs overall (BRCA 25.3%, CRC 14.8%, NSCLC 13.6%), lower than in syngeneic models. We delineated ten Mo/Mφ states: four monocyte subsets (Mo_CD14, Mo_CD16, Mo_ISG, and a Mo/T mixed population) and six macrophage subsets (Mφ_FOLR2, Mφ_PPARG, Mφ_SPP1, Mφ_MMP9, Mφ_TIMP3, and a proliferating Mφ_MKI67 state), each defined by distinct transcriptomes (**Figure 7D**, **Supplementary Figures S6F, G**). Monocytes accounted for 45.9% of the human Mo/Mφ compartment (BRCA 45.2%, CRC 53.4%, NSCLC 43.8%), exceeding the corresponding fraction in syngeneic models (**Figure 7E**).

In the macrophage compartment, a high SPP1+ and low C1QC+ TAM gene-signature combination has been reported to associate with poorer prognosis in CRC patients (10). In human tumors, Mφ_SPP1 and Mφ_PPARG displayed elevated M2-signature scores and high SPP1 expression, mirroring phenotypic features of mouse Mφ_Spp1_C10 and suggesting functional analogy (**Figure 7F**). Conserved markers included *FABP4*, *MMP12*, *ATP6V0D2*, and metallothionein genes (e.g., *MT1H*). ISG^high^ monocyte subsets were detected in both species with strong cross-species concordance; along with *IFIT1*/*2* and *ISG20*, these cells consistently expressed *TNFSF10*, *RSAD2*, and *CMPK2*, underscoring their translational relevance (**Figure 7G**, **Supplementary Table 14**).

Collectively, the cross-species conservation of immune cell states and transcriptional features underscored the utility of syngeneic models for mechanistic inference and biomarker development, reinforcing the translational importance of our comprehensive TIME profiling.

## Discussion

### Prior research and the novelty of this study

Murine syngeneic tumor models, which preserve intact immune systems, serve as foundational platforms for preclinical immunotherapy research. Their molecular and immunologic characteristics have been extensively characterized using conventional approaches, including flow cytometry, microarray profiling, and bulk RNA sequencing, to quantify immune cell populations and examine gene expression in immunoregulatory pathways during tumor progression or therapeutic intervention (4–7). More recently, scRNA-seq has enabled deeper resolution of cellular heterogeneity within these models. For instance, Kumar et al. explored ligand–receptor interactions across cell types, particularly tumor and stromal cells, in six models (LL2, B16F10, EMT6, CT26, MC38, Sa1N) (8). Bhatt et al. focused on T cells in CT26 and MC38 (9), while Zhang et al. profiled myeloid cells in MC38 and Renca (10). Qu et al. employed a bilateral tumor model with scRNA-seq to identify immune cell types predictive of response to avelumab in CT26 (11). Carpen et al. characterized the immune landscape of two triple-negative breast cancer (TNBC) models (4T1, EMT6) under baseline conditions and following chemotherapy, immunotherapy, or their combination (12). Building upon these foundational studies, we isolated immune cells to generate a high-resolution map of the TIME across a panel of widely used syngeneic models. Our study provides the first comprehensive, cross-model characterization of immune cell populations in murine syngeneic tumor models.

In this study, we validated previously reported immunologic patterns, such as the enrichment of NK cells in CT26 tumors and the predominance of Mo/Mφs in MC38 tumors, while uncovering novel molecular and phenotypic distinctions among immune cell subtypes across models. Our dataset enabled the identification and characterization of underexplored immune populations, offering deeper insight into the cellular complexity of tumor-infiltrating immune cells. Among these, γδ T cells expressing high levels of *Il17a*, were notably abundant in the 4T1 model. This subset may promote tumor progression by recruiting pro-inflammatory or immunosuppressive myeloid cells (39). Across NK cell populations, functional profiling revealed substantial heterogeneity, with GL261 tumors harboring a significantly higher fraction of activated NK cells. Of particular interest were adaptive NK cells (NKG2C^+^NKG2A⁻), known for their augmented cytokine responses and resilience to immunosuppression (30), which were predominantly found in GL261 tumors. Our analysis also expanded the understanding of DC diversity within syngeneic TIME. We identified a *Ccr7*+ DC population, previously shown to enhance antitumor CD8+ T cell responses through interleukin-12 secretion (40). Within the cDC2 compartment, transcriptional profiling revealed substantial functional heterogeneity. Subsets such as *Mgl2*+ cDC2s, associated with Th2 polarization and Tfh suppression (41, 42), and *T-bet*+ cDC2s, marked by high *Tbc1d4* expression and attenuated inflammatory potential (43), underscore the nuanced roles of DCs in orchestrating immune responses within the TME.

Our study further illuminated the functional complexity of Mo/Mφ and neutrophil populations in the context of immunotherapy. To investigate their roles in modulating antitumor responses, we first stratified Mo/Mφ subsets using canonical M1/M2 signatures and evaluated their association with PD-1 blockade efficacy across models. In MC38 tumors, which are characterized by limited T and NK cell infiltration, we observed an increased proportion of M1-like macrophages. This immunologic profile may underlie the model’s sensitivity to PD-1 inhibition. Although prior studies have linked *Cxcl9*-expressing macrophages to a favorable anti-PD-L1 response in CT26 (11), our data did not reveal consistent enrichment of this subset in responsive models. Instead, we identified a baseline elevation of an ISG^high^ monocyte population in models that responded to anti-PD-1 therapy. This observation aligned with a recent report demonstrating that inflammatory, ISG-enriched monocytes promote the expansion of tumor-specific CD8+ T cells and augment antitumor immunity, at the transcriptomic level, these monocytes were near-identical to our ISG^high^ subset (33). Debate regarding the role of TANs remains ongoing. For instance, neutrophils with elevated *SiglecF* expression are linked to tumor progression, and specific subsets, such as CCL4+ TANs and PD-L1+ TANs, recruit macrophages and suppress T cell cytotoxicity, respectively (38). Ng et al. demonstrated that diverse neutrophil populations infiltrate tumors but converge toward a pro-tumoral state (23). By contrast, ISG^high^ neutrophils accumulate during effective immunotherapy (35, 37). Notably, contrary to previous findings, such as those from Alb-Cre/Trp53fl/fl mouse models where neutrophil depletion reduced tumor progression (38), we observed model-specific effects. Neutrophil depletion exhibited a clear antitumor effect in the CT26.WT model but had no impact in the EMT6 model. Comprehensive profiling of neutrophil subsets and effector-molecule expression revealed model-specific polarization toward pro- or anti- tumor states. Moreover, the failure of neutrophil depletion to synergize with PD-1 blockade in either model, presumably owing to collateral ablation of the ISG^high^ neutrophil subset, further delineated functionally distinct neutrophil populations differentially contributing to immunotherapy-mediated tumor control. These findings underscore the importance of characterizing immune cell subsets and their functional states across tumor models, which is crucial for understanding the immune landscape of tumors and identifying targets to enhance cancer immunotherapy efficacy.

### Rational model selection and translational relevance

Our single-cell atlas of the TIME across widely used syngeneic models provided a comprehensive resource to support rational model selection and principled combination design. Models whose immune-cell composition and functional states most faithfully recapitulate the context of interest could be selected for immune-oncology studies. For instance, MC38 was enriched for inflammatory monocytes, whereas Renca and LL2 exhibited M2 polarization (LL2 dominated by SPP1+/ARG1+ TAMs and Renca by C1Q+ TAMs) (**Supplementary Figure S7A**, **Supplementary Table 15**). These distinct myeloid states, with clear therapeutic implications, could guide the rational deployment of agents in contexts most likely to reveal pharmacodynamic modulation. The atlas further delineated model-specific constraints on antitumor immunity, thereby informing the rational design and evaluation of combination strategies. For example, B16F10 exhibited sparse overall infiltration yet a disproportionately high fraction of CD8+ T cells, suggesting that bolstering T-cell abundance, preferably by driving proliferation rather than merely further attenuating inhibitory signaling in intratumoral T cells, could enhance responsiveness to anti-PD-1 therapy. As 4-1BB co-stimulation preferentially expands CD8+ T cells (44), our data were consistent with reports that 4-1BB plus PD-1, rather than LAG-3 plus PD-1, exhibited synergy in B16F10 melanoma (45).

Moreover, robust translational alignment in target and biomarker development is achievable through systematic interrogation of our murine single-cell profiles against human datasets. We curated and integrated human scRNA-seq data from treatment-naïve TIICs across 14 studies spanning diverse malignancies and benchmarked these against the murine TIME. In the human datasets, T cells constituted the predominant fraction of infiltrates, whereas Mo/Mφ were comparatively enriched in mice. Within the T-cell compartment, despite interspecies differences in subset composition and cell state, Tregs and CD8+ T cells exhibited conserved marker repertoires and functional programs. Across the myeloid compartment, orthologous marker genes were strongly concordant at the level of major lineages and across most Mo/Mφ sublineages. Conserved molecular signatures identified in SPP1+ TAMs and ISG^high^ monocytes further attested to the translational utility of syngeneic models. Collectively, these findings positioned our atlas as a preclinical analogue closely aligned with the human immune landscape, enabling rigorous prioritization of therapeutic targets and biomarker discovery in translational studies.

### Limitations and confounders

scRNA-seq is highly effective at resolving intra-compartmental heterogeneity and discriminating closely related subsets. However, RNA and protein abundance do not always exhibit a linear relationship at single-cell resolution, owing to both biological regulation and technical constraints. This discordance complicates the alignment of scRNA-seq-defined clusters with functional cell populations traditionally delineated by surface markers. Canonical markers such as CD3 for T cells, CD19 for B cells, and NKp46 for NK cells are robustly detected at the transcriptomic level, whereas others such as Ly6G for neutrophils are sparsely captured in scRNA-seq datasets. At the sublineage level, even within well-characterized T cell subsets, in which combinations such as CD62L and CD44 are routinely used to infer naïve, memory, or effector states, the transcript abundance of CD62L (*Sell*) does not consistently recapitulate its surface expression. To ensure reliable subpopulation delineation and functional annotation, we favored combinatorial marker schemes over single-gene readouts, integrating lineage-defining transcription factors with gene signatures anchored to established phenotypes. To further resolve the biological processes operative within each cell type, we conducted pathway enrichment analyses (**Supplementary Tables 8**–**12**) and applied automated annotation (**Supplementary Table 13**) to map transcriptional programs onto functional states. Nevertheless, multimodal experimental validation is required to ensure that the inferred states faithfully reflect biological reality.

It is important to acknowledge the variability between studies and datasets when evaluating immune-cell composition and ICB responses across syngeneic tumor models. For example, in our study, 4T1 and EMT6 were resistant to anti-PD-1 therapy, while MC38 was responsive. In contrast, Benguigui et al. reported a response in 4T1 (37), Jin et al., observed a response in EMT6 (46), and Mosely et al. found both 4T1 and MC38 to be non-responsive (4) to anti-PD-L1 therapy. These discrepancies may stem from differences in mutagenized clones (37), ICB agents, dosing regimens, treatment initiation time, or endpoint measurements (4, 46). Additionally, variations in implantation sites (subcutaneous vs. orthotopic), tumor inoculation volume and tumor-intrinsic features (**Supplementary Table 1**) can significantly influence immune landscape and, consequently, therapeutic outcomes. For example, the orthotopic 4T1 model exhibited an immune landscape similar to subcutaneous tumors but with greater variability in immune cell abundances (47). The number of cells used to establish tumors affects tumor latency, immune infiltration, and ICB responsiveness (48). In our scRNA-seq profiling, tumors were harvested at an average volume of 250–300 mm³, whereas some studies collected tumors at ~150 mm³, corresponding to the typical treatment initiation point in their efficacy studies (4). Despite these differences, our dataset exhibited strong concordance with previous datasets (**Figure 2D**, **Supplementary Figure S2C**), and each model displayed a reproducible, model-specific immune fingerprint across biological replicates, underscoring the robustness of the observed immune states. It is also worth noting that some prior scRNA-seq studies were conducted in genetically engineered mouse models, which may account for differences in immune composition. While our neutrophil subsets shared consistent marker gene expression with Zilionis’s study (34), their overall transcriptomic profiles were less congruent, potentially reflecting model-specific or technical variations.

In addition, interpreting the function of any immune-cell subset requires situating the evidence within the complex TME, where multicellular crosstalk and spatial architecture calibrate immune tone and responses to ICB. In our study, ISG^high^ monocytes were enriched in models responsive to anti-PD-1 therapy, and recent work has provided mechanistic insights into antitumor immunity (33), nominating them as putative predictive biomarkers and therapeutically actionable subpopulations. Nevertheless, ISG programs reflect a coordinated, multi-lineage activation. Given the extensive intercellular communication within the TME (**Supplementary Figures S7B, C**), ISG^high^ monocytes are more likely to act as components of the broader ecosystem rather than in isolation. As a consequence, confounding influences from other cell types cannot be readily excluded. For example, ISG^high^ CD8+ T cells may dampen the efficacy of anti-PD-1 therapy (49, 50), whereas ISG^high^ neutrophils have been associated with favorable responses to immunotherapy (35, 37). Caution is likewise warranted when inferring the contributions of individual cellular subsets from lineage-depletion experiments. For example, Ly6G-mediated neutrophil depletion not only eliminated protumor neutrophil subsets but also incidentally ablated ISG^high^ neutrophils associated with effective immunotherapy, thereby producing model-specific anti-tumor effects while precluding synergy with ICB. Consistent with these observations, previous studies have documented model-specific variation, revealed by systematic *in vivo* depletion of CD8+ T cells, CD4+ T cells, Tregs, NK cells, and macrophages, alone or in combination with anti-PD-1 (46). Taken together, across heterogeneous tumor models, antitumor activity and responses to anti-PD-1 vary, reflecting model-specific tumor-immune ecosystems and determinants of checkpoint sensitivity. Lineage-targeted and depletion strategies cannot assign causality to single populations due to extensive interactions among immune, malignant, and stromal compartments. The lack of selective tools for discrete subtypes further limits rigorous functional attribution. Additional mechanistic studies are needed to define intercellular crosstalk during immunotherapy.

## Data Availability

The data generated in this study are available at Gene Expression Omnibus (GEO) accession no. GSE307143.

## Supplementary material

Supplementary Figure 1 Quality control and data integration. **(A)** Number of cells harvested from each tumor model. **(B)** Boxplots illustrating the number of UMIs (left), genes (middle), and the proportion of mitochondrial DNA in cells (right) across various tumor models. **(C)** UMAP visualization of cell clustering categorized by models (left) or cell types (right) without the integration of CCA data. **(D)** Expression of hallmark genes utilized for the identification of major immune cell lineages, color-coded on UMAP without CCA data integration. **(E)** Bubble heatmap showing the expression levels of selected top signature genes in each cell type, as illustrated in **Figure 1B**. Dot size indicates the fraction of expressing cells, colored based on normalized expression levels.

Supplementary Figure 2 Cross-validation with published mouse model data. **(A)** UMAP plots of the reference dataset (GSE168944) with original annotations (left), projection of this study’s data onto the reference UMAP structure with transferred annotations (middle), and annotations from this study (right). **(B)** Projection of key T cell marker gene expression from this study onto the reference UMAP structure. **(C)** Heatmap showing the similarity between the reference data (GSE168944) and data from this study, annotated with transferred labels. **(D)** Proportions of T cell subtypes across datasets, based on GSE168944 annotations.

Supplementary Figure 3 Assessment of anti-PD-1 therapeutic efficacy across diverse syngeneic tumor models.

Supplementary Figure 4 Absolute fraction of Mo/Mφ subsets in syngeneic tumors. **(A)** Representative images of Immunohistochemistry (IHC) staining for CD45 in synthetic tumor samples. Brown color indicates positive staining. (Scale bar = 2 mm). **(B)** Correlation between *Cd45* expression levels and immune cell fractions estimated via quanTIseq from bulk RNA-seq data. **(C)** Boxplots of estimated immune cell fractions across tumor models. **(D)** Fraction of each Mo/Mφ subset in anti-PD-1 responsive or resistant tumors, with mean values used for each model.

Supplementary Figure 5 Functional characterization of tumor-infiltrating neutrophils. **(A)** Gene Ontology (GO) enrichment analysis of differentially expressed genes for each neutrophil subtype, shown as a dot plot (x-axis: enrichment score; y-axis: GO terms). **(B)** Violin plot illustrating signature scores for neutrophil maturation and T1, T2 gene signatures as defined by Ng et al. **(C)** Comparative transcriptomic analysis of neutrophils from this study and published data (GSE127465). **(D)** Proportion of neutrophils among live CD45+ cells in CT26.WT and EMT6 models following Ly6G-mediated depletion. **(E)** Grouped box plots showing the percentage of each neutrophil subset within the neutrophil compartment in CT26.WT and EMT6 models. **(F)** Bubble heatmap displaying the expression of anti- and pro-tumor factors across neutrophil subsets in CT26.WT and EMT6 models.

Supplementary Figure 6 Single-Cell Transcriptional Profiling of public Human datasets. **(A)** Bubble heatmap of T/NK cell subsets and cluster-specific marker genes. **(B)** Heatmap showing cross-species concordance of marker gene expression patterns across major T-cell and myeloid lineage states. **(C)** UMAP projection of principal myeloid lineages from public human single-cell RNA-seq datasets. **(D)** Bubble heatmap of genes marking principal myeloid populations. **(E)** Compositional profiles of principal myeloid cell types, reported as fractions within the myeloid compartment. **(F, G)** For Mo/Mφ subsets, bubble heatmap of selected top marker genes **(F)** and corresponding signature scores **(G)** across subsets.

Supplementary Figure 7 Distinct Cellular and Chemokine Landscapes in Syngeneic Tumor Models **(A)** Cellular architecture of the TIME across syngeneic models, delineated by hierarchical clustering of cell subtype abundance. The color gradient reflects the normalized relative abundance of each cell subtype within the TIME. **(B, C)** Heatmaps illustrating RNA expression levels of chemokines and their corresponding receptors in bulk tumor samples **(B)** and in single-cell TIIC subpopulations **(C)**.
