# Supplementary figures and images for: Single-cell atlas of the tumor immune microenvironment across syngeneic murine models
Source: Front Immunol. 2025 Nov 14;16:1676581. doi: 10.3389/fimmu.2025.1676581 (PMC12660277; doi:10.3389/fimmu.2025.1676581)

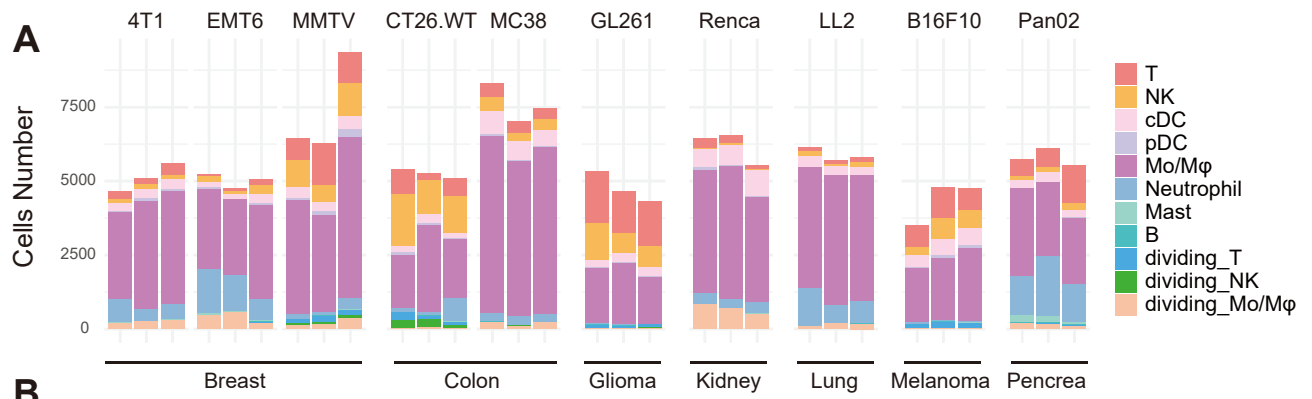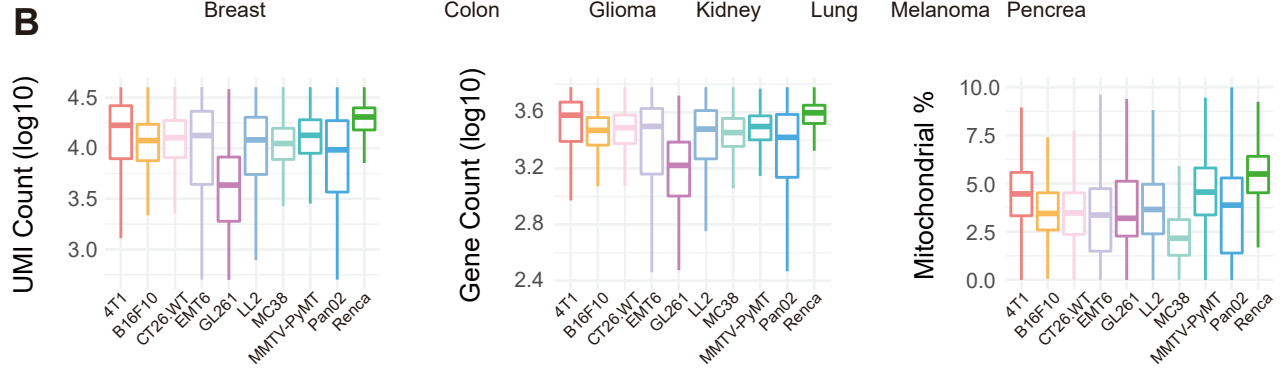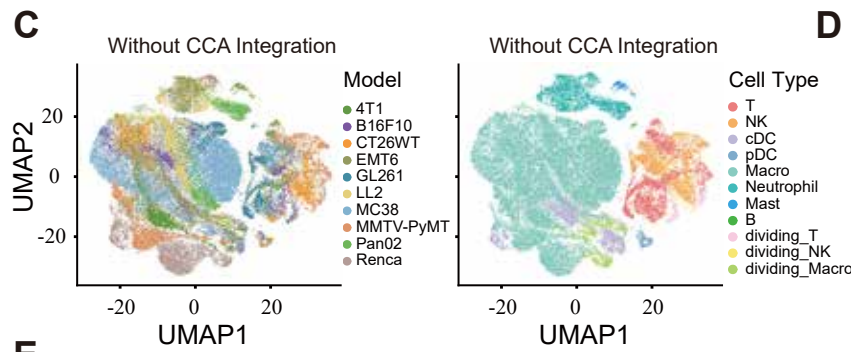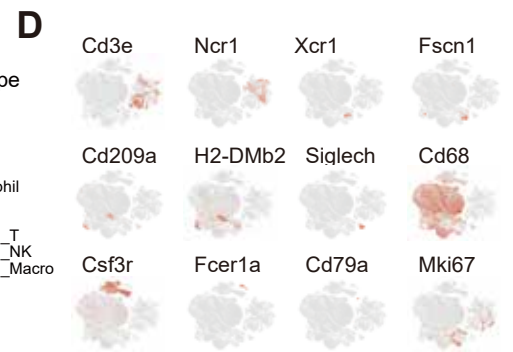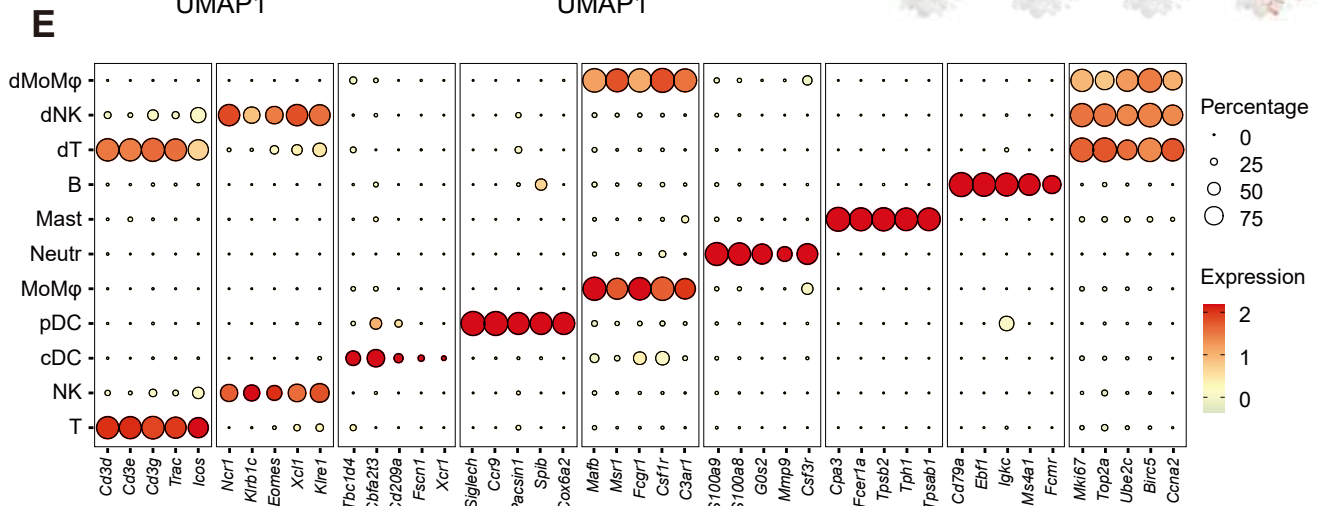

Supplement: Supplementary Figure 1 — Quality control and data integration. (A) Number of cells harvested from each tumor model. (B) Boxplots illustrating the number of UMIs (left), genes (middle), and the proportion of mitochondrial DNA in cells (right) across various tumor models. (C) UMAP visualization of cell clustering categorized by models (left) or cell types (right) without the integration of CCA data. (D) Expression of hallmark genes utilized for the identification of major immune cell lineages, color-coded on UMAP without CCA data integration. (E) Bubble heatmap showing the expression levels of selected top signature genes in each cell type, as illustrated in Figure 1B. Dot size indicates the fraction of expressing cells, colored based on normalized expression levels. [file Image1.pdf]

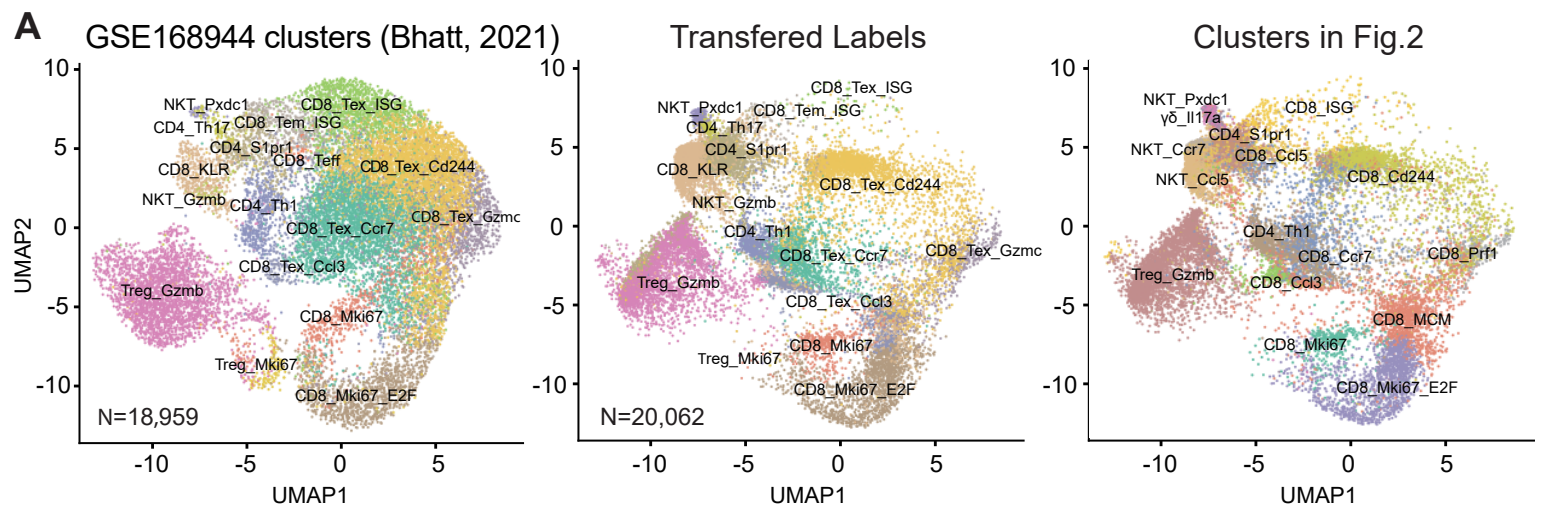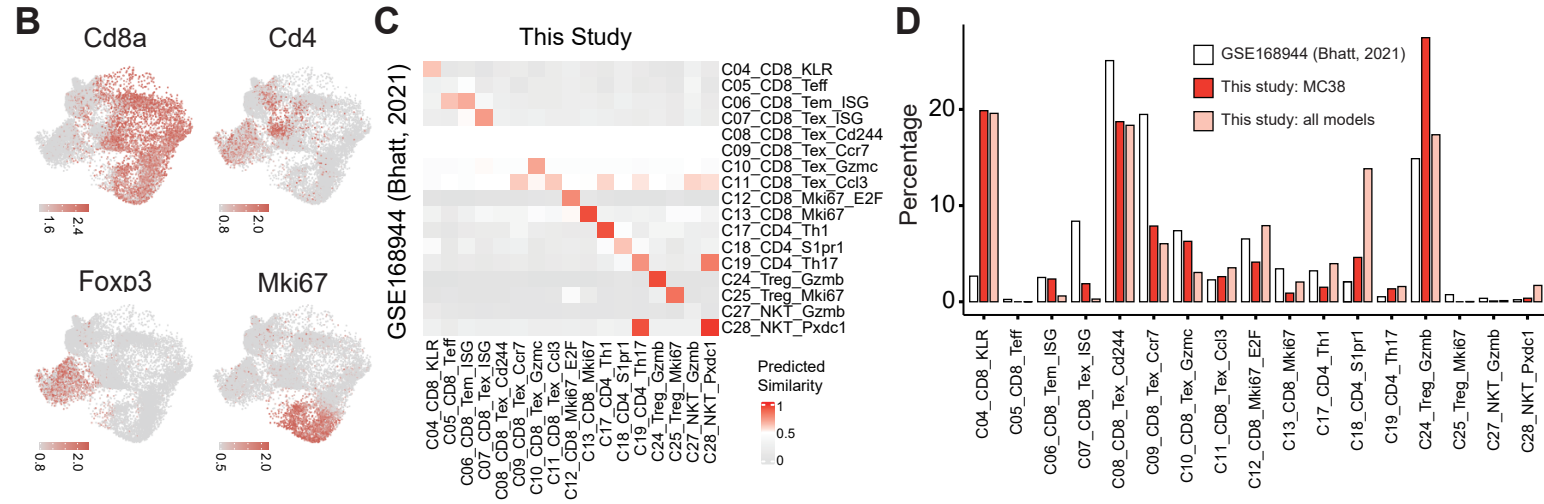

Supplement: Supplementary Figure 2 — Cross-validation with published mouse model data. (A) UMAP plots of the reference dataset (GSE168944) with original annotations (left), projection of this study’s data onto the reference UMAP structure with transferred annotations (middle), and annotations from this study (right). (B) Projection of key T cell marker gene expression from this study onto the reference UMAP structure. (C) Heatmap showing the similarity between the reference data (GSE168944) and data from this study, annotated with transferred labels. (D) Proportions of T cell subtypes across datasets, based on GSE168944 annotations. [file Image2.pdf]

## PD-1 sensitive models

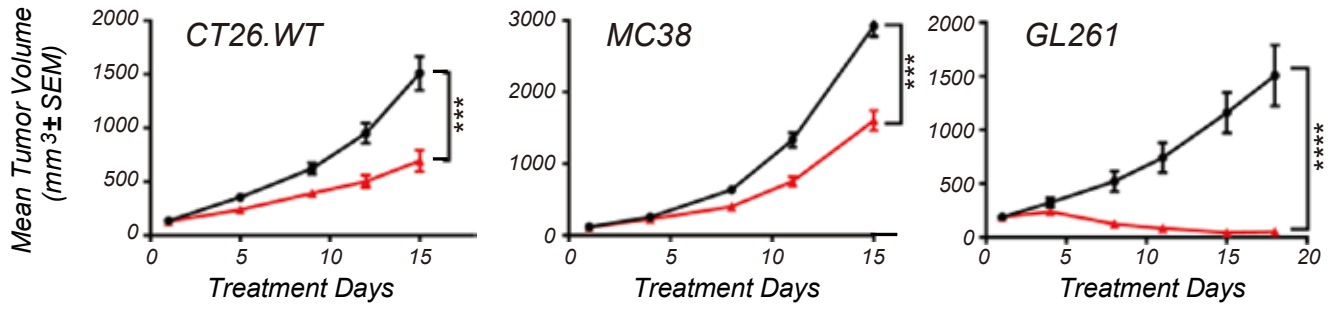

## PD-1 insensitive models

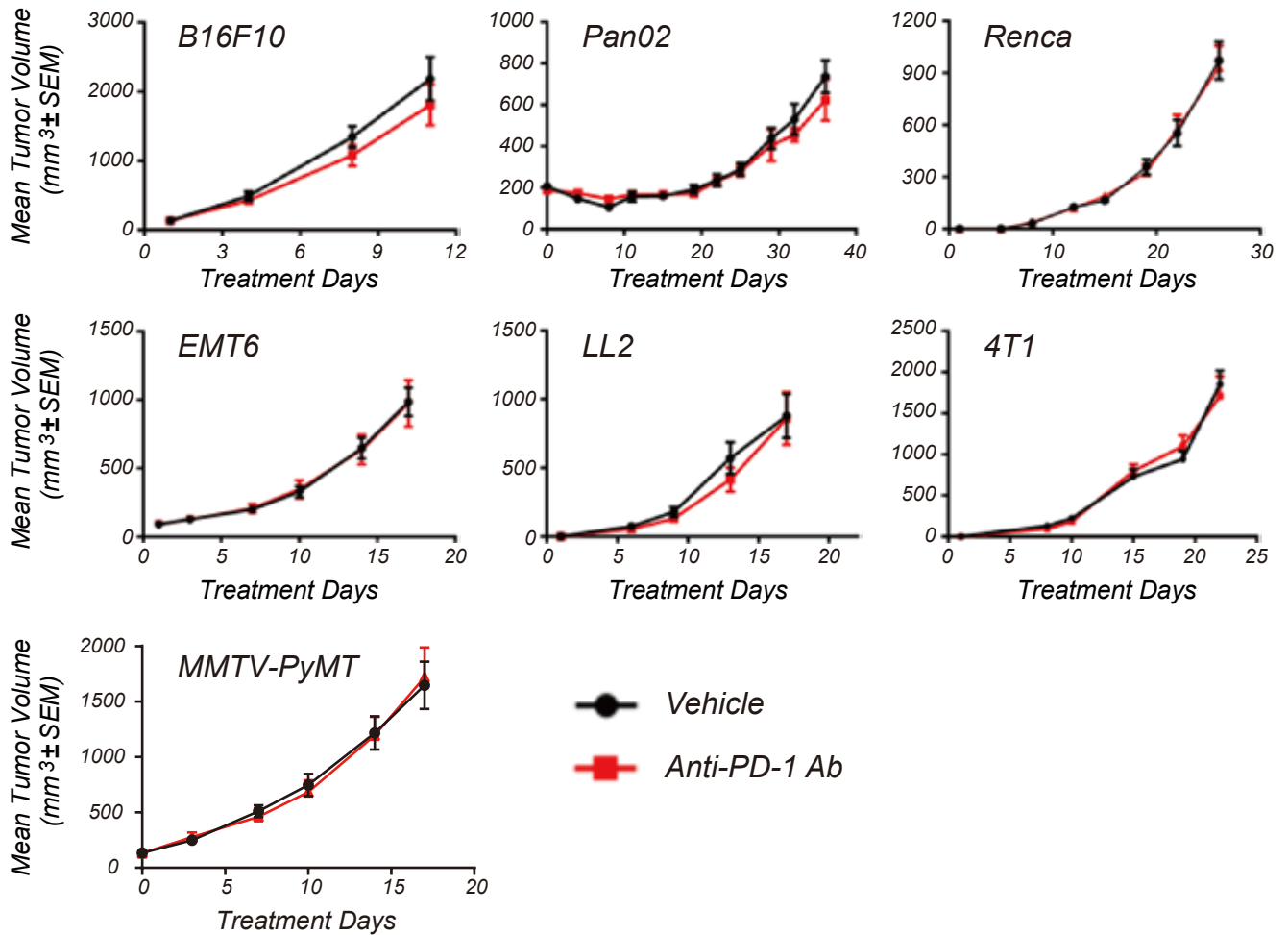

Supplement: Supplementary Figure 3 — Assessment of anti-PD-1 therapeutic efficacy across diverse syngeneic tumor models. [file Image3.pdf]

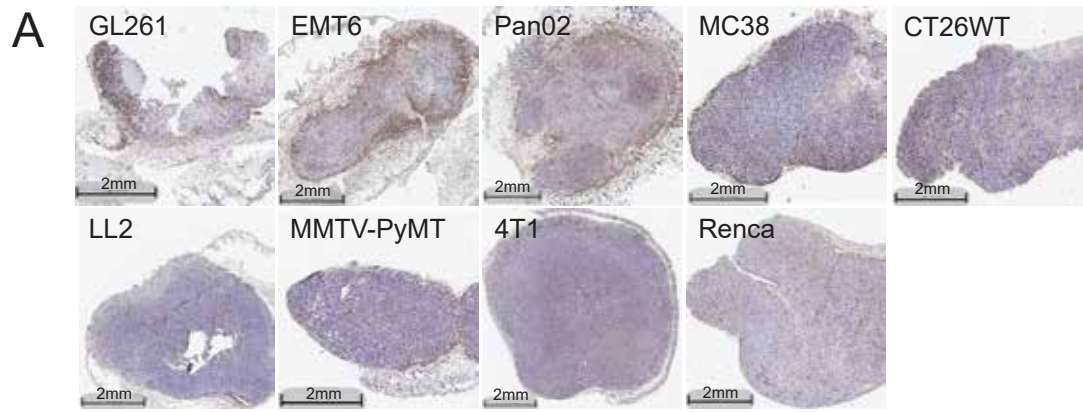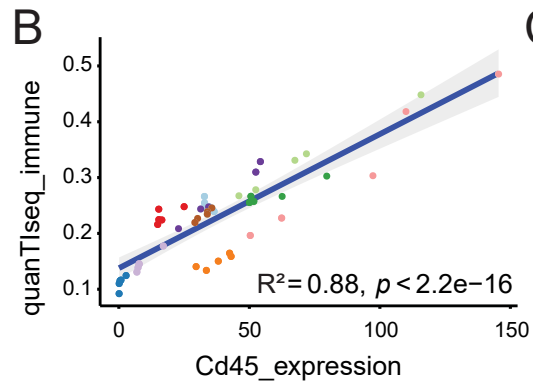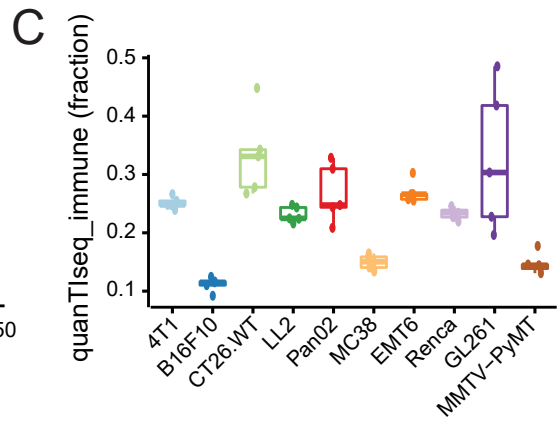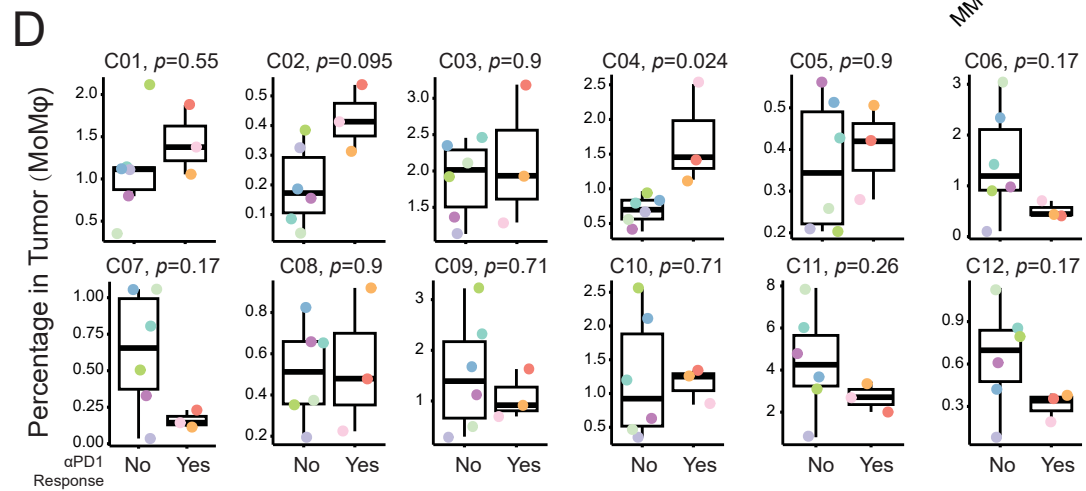

Supplement: Supplementary Figure 4 — Absolute fraction of Mo/Mφ subsets in syngeneic tumors. (A) Representative images of Immunohistochemistry (IHC) staining for CD45 in synthetic tumor samples. Brown color indicates positive staining. (Scale bar = 2 mm). (B) Correlation between Cd45 expression levels and immune cell fractions estimated via quanTIseq from bulk RNA-seq data. (C) Boxplots of estimated immune cell fractions across tumor models. (D) Fraction of each Mo/Mφ subset in anti-PD-1 responsive or resistant tumors, with mean values used for each model. [file Image4.pdf]

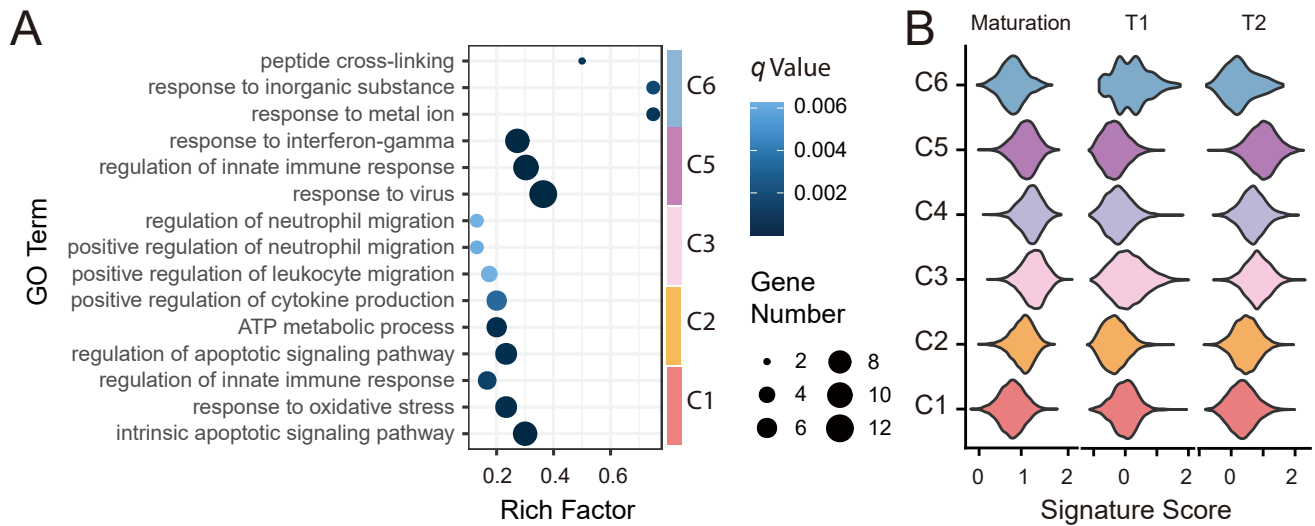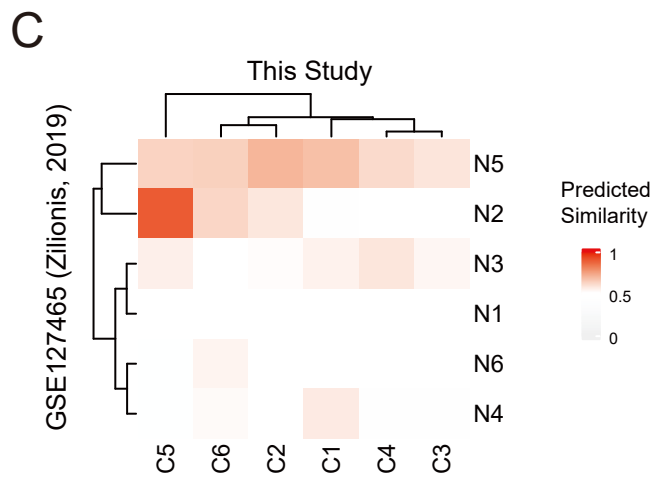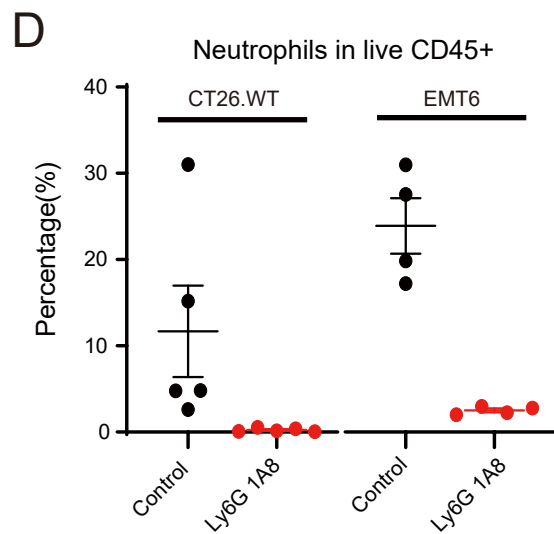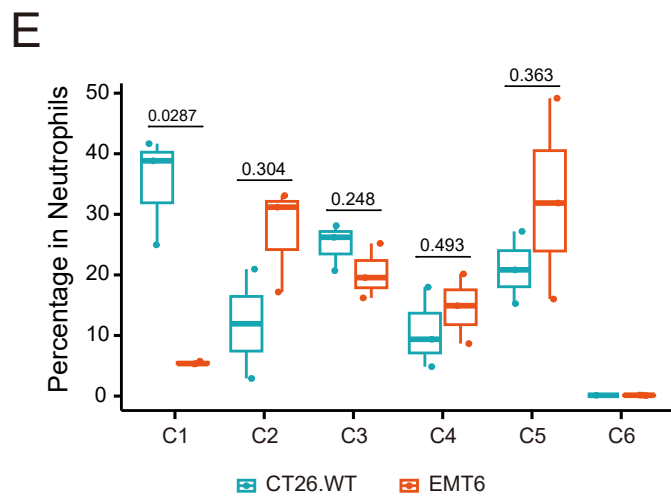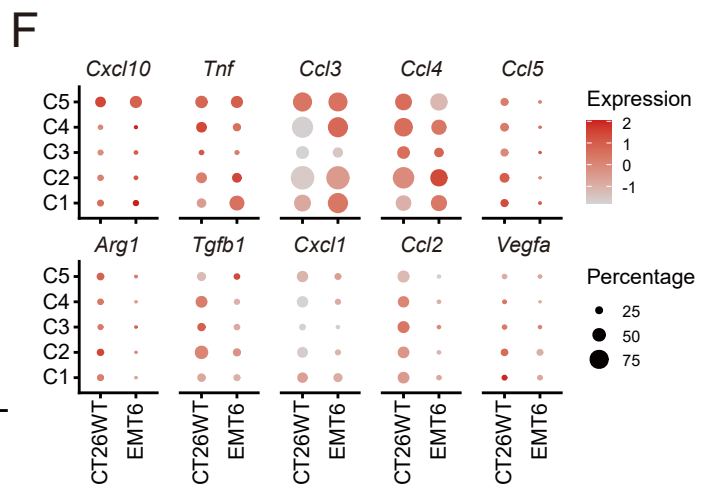

Supplement: Supplementary Figure 5 — Functional characterization of tumor-infiltrating neutrophils. (A) Gene Ontology (GO) enrichment analysis of differentially expressed genes for each neutrophil subtype, shown as a dot plot (x-axis: enrichment score; y-axis: GO terms). (B) Violin plot illustrating signature scores for neutrophil maturation and T1, T2 gene signatures as defined by Ng et al. (C) Comparative transcriptomic analysis of neutrophils from this study and published data (GSE127465). (D) Proportion of neutrophils among live CD45+ cells in CT26.WT and EMT6 models following Ly6G-mediated depletion. (E) Grouped box plots showing the percentage of each neutrophil subset within the neutrophil compartment in CT26.WT and EMT6 models. (F) Bubble heatmap displaying the expression of anti- and pro-tumor factors across neutrophil subsets in CT26.WT and EMT6 models. [file Image5.pdf]

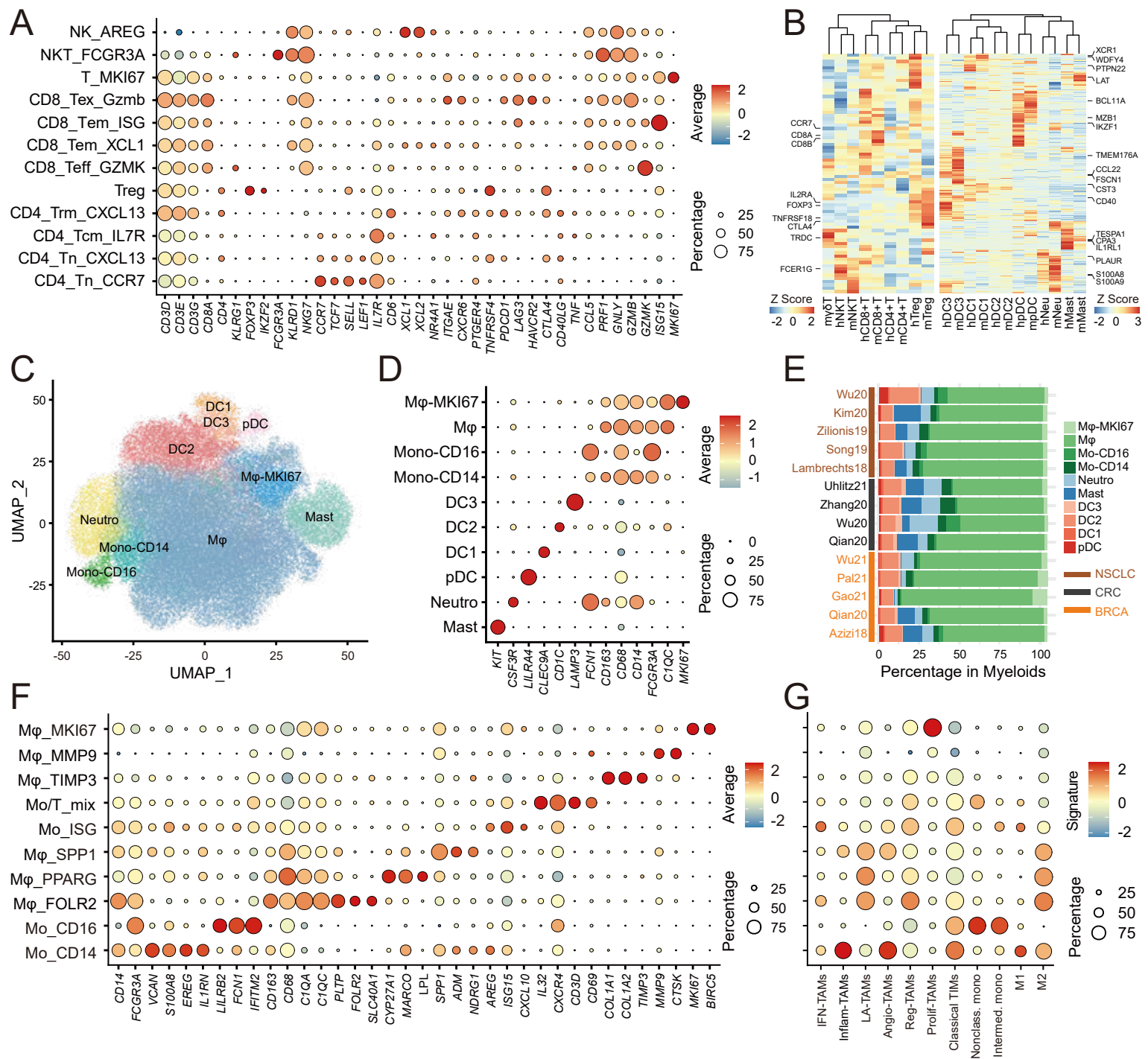

Supplement: Supplementary Figure 6 — Single-Cell Transcriptional Profiling of public Human datasets. (A) Bubble heatmap of T/NK cell subsets and cluster-specific marker genes. (B) Heatmap showing cross-species concordance of marker gene expression patterns across major T-cell and myeloid lineage states. (C) UMAP projection of principal myeloid lineages from public human single-cell RNA-seq datasets. (D) Bubble heatmap of genes marking principal myeloid populations. (E) Compositional profiles of principal myeloid cell types, reported as fractions within the myeloid compartment. (F, G) For Mo/Mφ subsets, bubble heatmap of selected top marker genes (F) and corresponding signature scores (G) across subsets. [file Image6.pdf]

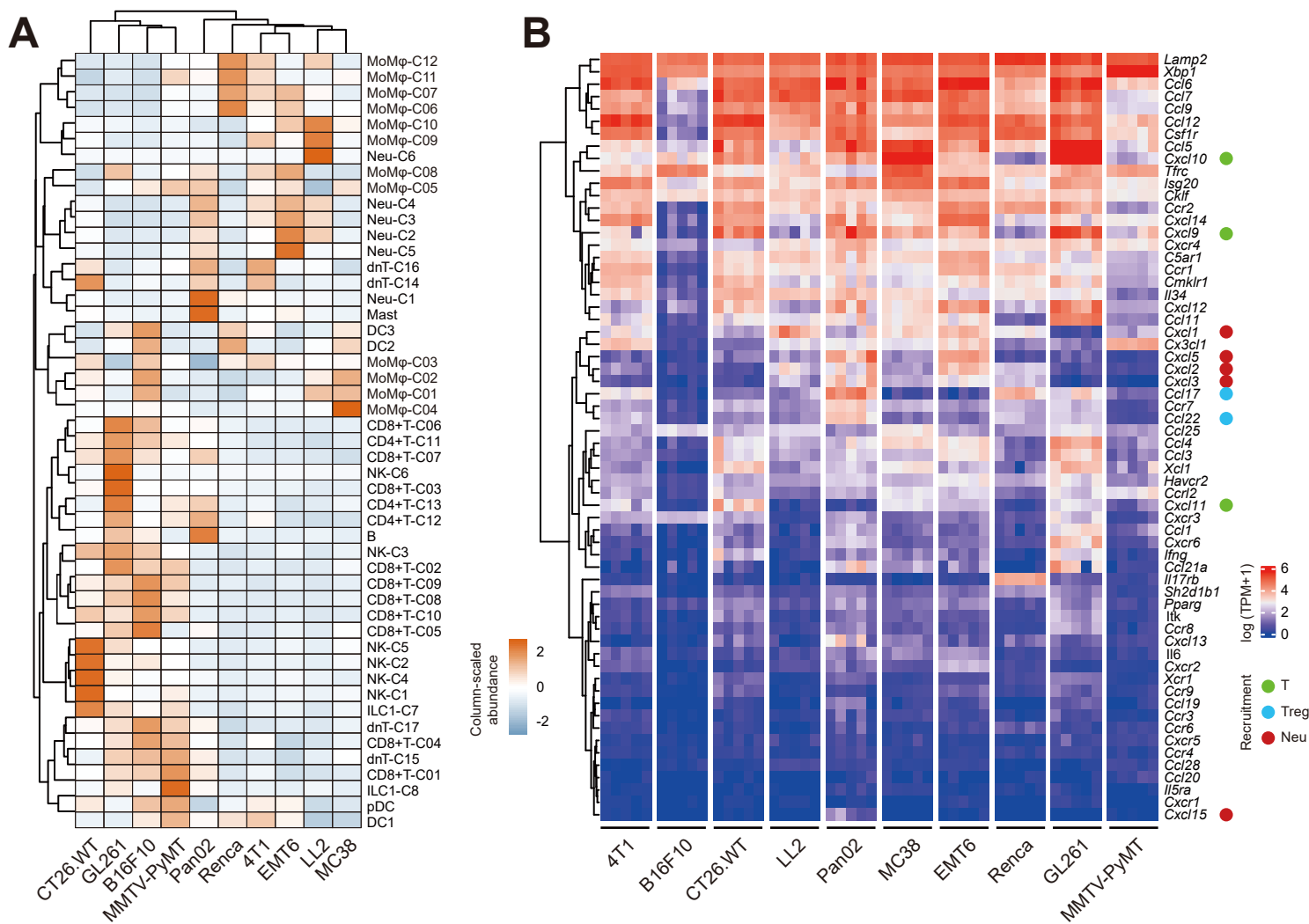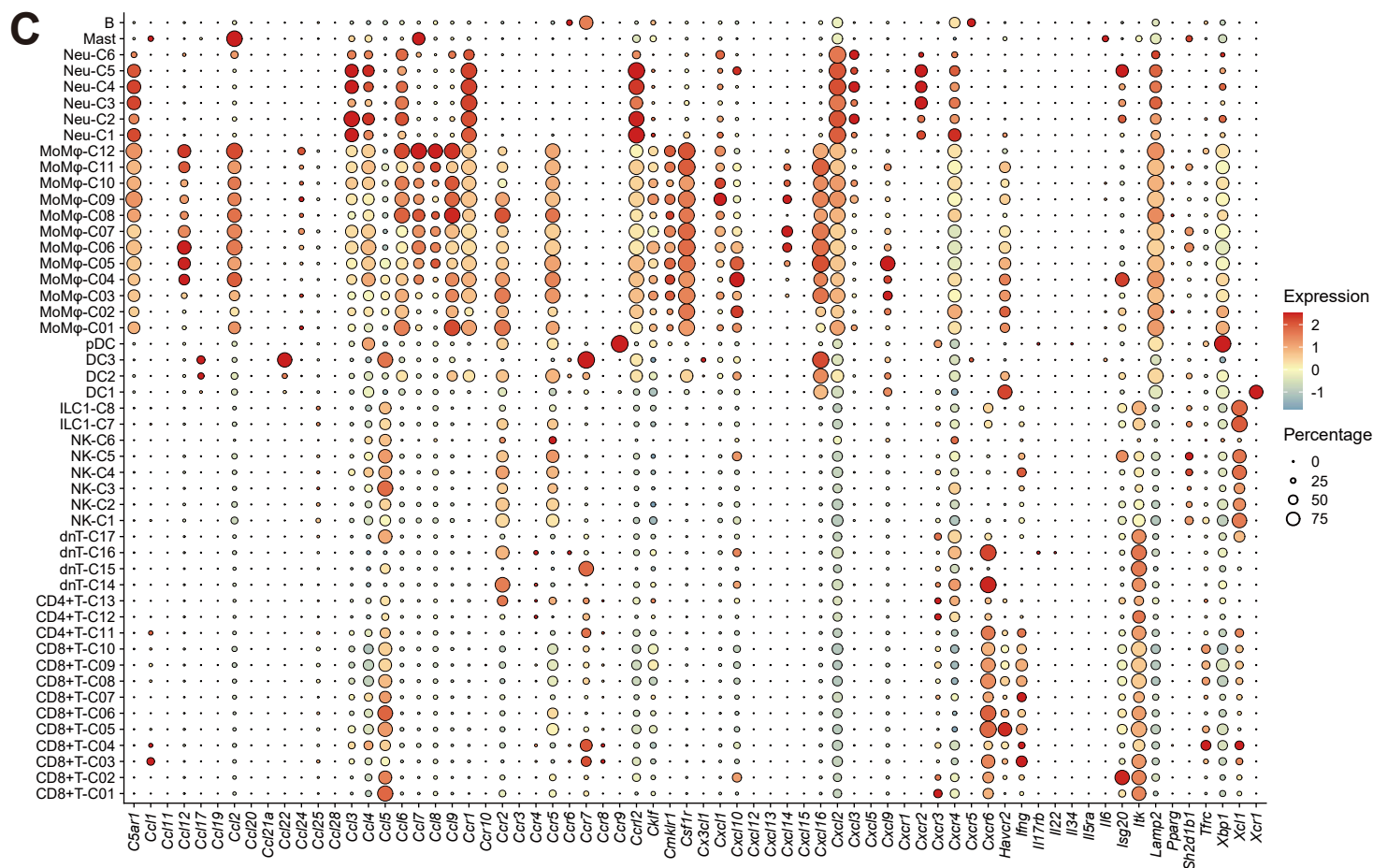

Supplement: Supplementary Figure 7 — Distinct Cellular and Chemokine Landscapes in Syngeneic Tumor Models (A) Cellular architecture of the TIME across syngeneic models, delineated by hierarchical clustering of cell subtype abundance. The color gradient reflects the normalized relative abundance of each cell subtype within the TIME. (B, C) Heatmaps illustrating RNA expression levels of chemokines and their corresponding receptors in bulk tumor samples (B) and in single-cell TIIC subpopulations (C). [file Image7.pdf]
